# A comparative evaluation of maize silage quality under diverse pre-ensiling strategies

**DOI:** 10.1371/journal.pone.0308627

**Published:** 2024-09-18

**Authors:** Lorenzo Serva

**Affiliations:** Department of Animal Medicine, Production, and Health, University of Padova, Padova, Italy; Universidad de Costa Rica, COSTA RICA

## Abstract

Maize silage serves as a significant source of energy and fibre for the diets of dairy and beef cattle. However, the quality of maize silage is contingent upon several crucial considerations, including dry matter loss, fermentative profile, pH level, ammonia content, and aerobic stability. These aspects are influenced by a multitude of factors and their interactions, with seasonality playing a crucial role in shaping silage quality. In this study an open-source database was utilised to assess the impact of various pre-ensiling circumstances, including the diversity of the chemical composition of the freshly harvested maize, on the silage quality. The findings revealed that seasonality exerts a profound influence on maize silage quality. Predictive models derived from the composition of freshly harvested maize demonstrated that metrics were only appropriate for screening purposes when utilizing in-field sensor technology. Moreover, this study suggests that a more comprehensive approach, incorporating additional factors and variability, is necessary to better elucidate the determinants of maize silage quality. To address this, combining data from diverse databases is highly recommended to enable the application of more robust algorithms, such as those from machine learning or deep learning, which benefit from large data sets.

## Introduction

Maize (*Zea mays* L.) silage is a major forage and energy source in dairy and beef cattle nutrition across numerous countries, including Italy [1]. Maize silage typically comprises 25%–35% starch and 40%–50% neutral detergent fibre (**NDF**) [2]. As maize is the main roughage ingredient in the total mixed ration (**TMR**) for dairy and beef cattle [3], it is crucial to understand and evaluate its characteristics. Maize silage has been extensively studied with regard to its chemical, microbial, and organoleptic traits and its dry matter (**DM**) and quality loss during the ensiling process [1, 4, 5]. However, fermentation parameters can influence the DM intake and, consequently, the milk yield, the energy-corrected milk, and milk fat, and protein [6, 7] contents. Several factors may define the silage quality—including the pH and the concentration of a complex array of compounds, such as ammonia, lactic, acetic, propionic and butyric acids, alcohols, and esters [5]—and the composition in terms of DM such as crude protein (**CP**), starch, fibre fractions, and nutrient digestibility [8–10]. To improve data interpretability, the maize silage fermentative quality was represented by a fermentation quality index (**FQI**) [11, 12] as well as integrated with nutritional parameters and measured milk yield [7, 13]. However, high-quality silage should be void of undesirable compounds that could negatively affect animal performance, environment, or net farm income. Additionally, silage quality is related to its aerobic stability, commonly defined as the number of hours that the silage remains stable before reaching 2°C above the ambient temperature [14–16]. Finally, the maize silage is subject to DM and quality losses that occur during the ensiling process from the field through the feeding phase [1].

The fermentation products in silage are strongly affected by the characteristics of the fresh-ensiled plant, such as DM, protein, fibre, N-free extract and water-soluble carbohydrates (**WSC**) [5, 17, 18] content. Therefore, the maize silage quality may be assessed beginning from the characteristics of the fresh plant [19]. Although maize silage quality is also the result of several factors that are present before ensiling—such as the use of inoculants [20, 21], the short time delayed sealing [22, 23], maturity stage at harvest [4, 24, 25], maize hybrid [3, 4, 12], silos compaction [26], and use of oxygen barrier film for covering silos [27], the aerobic exposure after silo opening (feed-out phase) is relevant after ensiling [26], including the feed-out rate [1].

Further, bacterial inoculants have been previously employed to mitigate variations stemming from the natural epiphytic bacterial populations and the composition of forage that can occur during the ensiling process. Typically, they achieve this by expediting the decrease in pH after ensiling and enhancing the preservation of DM and nutrients [28]. In silage, the lack of oxygen and the presence of lactic acid—which lowers pH—inhibits undesirable microbial metabolism and preserves nutrients. When exposed to air, certain opportunistic microorganisms (yeasts and moulds) become metabolically active, producing heat, and consuming nutrients from the silage, thereby resulting in spoilage and aerobic deterioration [15]. Silage preservation relies on the absence of oxygen and the instauration of the acidification process, which occurs when the natural lactic acid bacteria (**LAB**) population on the surface of the plant generates an adequate quantity of lactic acid during harvesting [29]. Inoculants for silage have been the most frequently employed additive for enhancing silage quality [30]. To this end, inoculation with selected homolactic and facultative heterolactic LAB ensure a rapid and efficient fermentation of WSC into organic acids, rapidly produce lactic acid and lower pH to help improve the efficiency of the fermentation process, and reduce DM losses [31]. Indeed, heterofermentative LAB, particularly *Lentilactobacillus buchneri* (**Lb**), known to thrive in the later stages of fermentation, exhibit increased production of acetic acid [32, 33], which possesses the capability to inhibit yeasts responsible for prompting aerobic spoilage.

A complete consensus among researcher on the effectiveness of corn silage inoculants in improving silage quality has yet to be reached, and it has been reported that the responses of forages to inoculants during the ensiling process could be inconsistent [8]. Further, a meta-analysis also revealed that inoculating with homofermentative or facultative heterofermentative LAB (applied at a rate of ≥ 10^5^ CFU × g^-1^ as fed) had a positive impact on temperate and tropical grasses as well as on alfalfa and other legume silages. However, this treatment did not improve corn, sorghum, and sugarcane silage fermentation [34]. Moreover, a meta-analysis conducted from 1980 to 2017 indicated that the impact of inoculants may vary depending on whether homofermentative or heterofermentative LAB is utilized. Therefore, in developing effective bacterial inoculants, it is advisable to incorporate both types of LAB [35]. Although numerous studies have demonstrated the positive effects of LAB inoculation on fermentation characteristics [35–37]—including the domination of the epiphytic microbial population and consequent improvements in fermentation, shelf life, and silage quality [38]—it is essential to note that a range of factors can influence the outcomes associated with silage inoculants. These factors encompass the duration of the ensiling process, the application rate of the LAB [14, 15] inoculant, the specific LAB species utilised [29], the use of forage-specific inoculants related to the ensiling ability of forage [8, 14], and other aspects related to silage management practices [39]. Therefore, comprehensive studies that include all these possible covariates are required.

Directly sharing data with other researchers or through personal data storage has become common [40]. However, when data sets are easily accessible, researchers are generally more willing to reuse data provided by others. A recent survey [40] found that 54.3% of the respondents had reused existing data sets. Notably, the experience of data reuse varied significantly across different subject areas. With regard to the purposes of data reuse, researchers indicated that they combine multiple existing data sets to address novel research questions (63.1%), compare or ground truthing (50.7%), and answer new research questions (46.6%). Among those who had reused data sets, 60.9% discovered data sets by reading relevant research papers.

Based on the previous consideration, while numerous studies have been completed under restrictive design and aimed to address specific issues, a broader and untargeted approach can be employed by merging several data sets to find a more comprehensive interaction among factors influencing silage quality.

Recently, a dataset [41] has been released with the principle of data sharing among researchers. This dataset comprises seven years’ worth of data collected across various trials, all aimed at evaluating the quality of maize silage under different pre-ensiling conditions. This data set could be merged with other researchers’ data to enhance a large dataset capable of comprehensively understanding the ensiling process and fermented preservation.

In particular, in the assessment of the effectiveness of new additives—such as inoculants—and their combinations, laboratory-scale silos are initially employed. The high efficiency of these smaller-scale systems, including vacuum bags or fixed-volume vessels, provides valuable insights that could guide the design of more resource-intensive and labour-intensive farm-scale studies; these methods have all been shown to be suitable for modelling larger-scale ensiling systems [42].This study aims to offer insights into the impact of various pre-ensiling conditions, including the use of inoculants at varying doses, on the quality of maize silage. This study is based on an earlier data set and is conducted under laboratory-scale conditions. Further, this study proposes that researchers should effectively combine and analyse this data to enhance predictive accuracy in their research.

## Materials and methods

### Description of the utilised data set description

When the terms “mazie” OR “corn” AND “silage” were typed in Google Dataset Search (data accessed 18/09/2023), Data (MDPI), and Data in Brief (Elsevier), the data search results did not allow other data sets to show up other than the proposed; therefore, to the best of our knowledge, there are not dataset available for this topic.

Thus, data were taken from a freely available database [43], whose geographic coverage is north-east Italy, and for which data were collected for over seven years (2016–2022). The data set comprises three trials (named #1, #2, and #3), all of which group data by adopted methods. The data from trials one to three were separately analysed and published elsewhere [11, 12, 19, 41, 44, 45], but the entire dataset has never been analysed earlier. Only the crucial aspects of the materials and methods are presented on this manuscript, while these studies can be referred to [11, 12, 19, 41, 44, 45] for more in-depth details.

Trial #1 was conducted in the Veneto region (northeast Italy) using 37 maize hybrids of early (**EA**; FAO class 200, n = 19) and late (**LA**; FAO class 600–700, n = 18) maturing classes for the period 2016–2019. Each hybrid was harvested in up to three plots, corresponding to three areas (level of input field, **IF**), which refers to soil fertility defined as ‘low’ (**IFL**) ‘medium’ (**IFM**), and ‘high’ (**IFH**). For each plot, every hybrid was harvested twice in two different subplots. EA and LA hybrids were sown at densities of 95,000 and 70,000 plants per ha, respectively. For each plot and subplot, freshly harvested maize (**FHM**) was sampled at three phenological maturity stages (**MSe**): early (**EH**; 1/3 milk line phase), medium (**MH**; at 2/3 milk line phase), and late harvest (**LH**; 5 d after the 2/3 milk line phase). For each plot, subplot, and maturity phase, approximately five plants were harvested, chopped, and mixed to obtain one sample. Each sample was split into two subsamples and analysed twice with near-infrared (NIR) spectroscopy, but the averages of scans and subsamples were performed before statistical analysis. Subsamples were promptly ensiled in vacuum-packed bags (Orved 2633040, Orved SpA, Musile di Piave, VE, Italy) for 60 days in a dark room with a stable temperature(23 ± 1°C).

Trial #2 comprised Trial #2.a (Trial #2.a.1.a, Trial #2.a.1.b., and Trial #2.a.2) and Trial #2.b. Trial #2.a was conducted in the Veneto region in a single IF (Lonigo: 45° 23’ lat. N, 11° 23’ long. EST; not estimated fertility, **IFN**), while Trial #2.b was conducted across the Veneto region (IF, unknown).

Trial #2.a tested different bacterial inoculant doses applied to the FHM of LA hybrid (KWS Kelindos, FAO class 600–130 d). FHM plants were ensiled by testing three mixtures of obligate heterofermentative (**He**) Lb CCM 1819 (KWS Lactostability, AGRAVIS Raiffeisen AG, Munster, Germany) at three different concentrations (CFU g^-1^ of FHM) and a mixture of homo-fermentative (**Ho**) lactic acid bacteria (*Lactiplantibacillus plantarum* NCIMB 30083–1k207736, *Lactiplantibacillus plantarum* NCIMB 30084–1k207737, *Peditococcus pentosaceus* DSM 23688–1k1010, *Peditococcus pentosaceus* DSM 23689–1k1019, and *Enterococcus faecium* 22502–1k20602) at a standard dose (**SD**) of 3 × 10^5^ CFU g^-1^ of FHW. The doses used for the inoculation with He were standard SD = 2.02 × 10^5^, half dose (**HD**) = 1.01 × 10^5^ CFU g^-1^, and double dose (**DD**) = 4.04 × 10^5^ CFU g^-1^. The control (**C**) was pure water. The inoculation was performed in a large sterile container, thereby enabling adequate mixing. For each tested mixture of inoculants (**MXe**), silages were prepared with delays of 0 h, 6 h and 20 h from the harvest time (**D0**, **D6,** and **D20**, respectively) to evaluate the delay effect (**DLe**). In 2029, the very late (VLH = 5 days after LH) will be added to the MSe thesis.

Moreover, Trial #2.a was arranged in Trial #2.a.1.a (in buckets, simulating the feed-out rate), Trial #2.a.1.b (in buckets, without simulating the feed-out rate), and Trial #2.a.2 (in bags). The samples from Trial #2.a.1.a and Trial #2.a.1.b were ensiled in a 20 L circular truncated conical plastic bucket and pressed with the use of a 1-tonne hydraulic press (141 kg cm^-2^) with the ideal purpose of reaching a density of 225 kg DM m^-3^ of ground FHM [46]. The buckets were sealed using a 150 μm SealPlus Film permeable to oxygen at a daily rate of 48 cm^3^ m^-2^ at 23°C and 65% RH (SealPlus by Gamma Srl, Mondovi, Italy) and with robust tape. The sealed buckets were stored in a dark room for 60 days at a stable temperature of 23 ± 1°C, thereby ensuring better anaerobic ensiling conditions. To simulate the feed-out rate (Trial #2.a.1.a), after removing 15 cm of eventually spoiled silage at the top of the buckets (**Spl**), the remaining silage was ideally divided into three layers in depth. At three opening times (**OT**), the layers were removed from the bucket and analysed immediately (day 1, **OT1**), after 48 h (day 3, **OT3**), and after 96 h (day 5, **OT5**). The buckets with the remaining parts were left in a dark, temperature-stable room. A data logger was repositioned 7.0 cm under the silage surface of OT1, OT3, and OT5 and recorded the temperature every 15 min with a precision of 0.1°C (Elitech USB Temperature Datalogger RC-5, London, UK). The data logger was repositioned 7 cm below the silage surface at each sampling time. The sealed buckets opened without intending to simulate the feed-out rate (Trial #2.a.1.b) and consisted of a single layer. After removing 15 cm of eventually spoiled silage and 1.0 kg of maize silage submitted to NIR analysis, the remaining portion was placed in an open and square polystyrene pan, with a capacity of 20 L and dimensions of 495 × 295 × 140 mm. A data logger was positioned 7 cm below the silage surface of the 20 L polystyrene pan and recorded the temperature every 30 min. In Trial #2.a.1.b in 2022, maize was sown in June and harvested in September–October, while in 2021 two pre-ensiled densities (**DEN**) were tested to attain the final low point (**DENl** of approximately 140 kg DM m^-3^) and high point (**DENh** of approximately 200 kg DM m^-3^).

Trial #2.a.2 tested 60 FHM samples ensiled using vacuum bags to which 5 MXe (3 He + 1 Ho + C) × 1 DLe × 4 MSe × 3 replicates were applied.

Trial #2.b tested operative scenarios sampling FHM in commercial farms during their harvesting activity and ensiling samples in the 20 L buckets, regardless of the use of inoculants, sealing delay (always considered at zero hours), and maturity stage at harvest. After 60 days of ensiling, the buckets were opened and samples simulated the feed-out rate (Spl, OT1, OT3, and OT5)

For clarity, Table 1 reports the tested effects for each trial.

**Table 1:** The tested effects in the trials defined in the dataset.

	Trial #1	Trial #2.a.1.a	Trial #2.a.1.b	Trial #2.a.2	Trial #2.b
Tested effects	2016–2019	2018–2020	2020, 2022	2019	2018, 2019
**Method**	Bags	Buckets (fos)	Buckets	Bags	Buckets (fos)
**Inoculant**		C, He, Ho	C, He, Ho	C, He, Ho	
**Dose**		HD, SD, DD	SD	HD, SD, DD	
**Delay**	none	D0, D6, D20	D0, D6	D0	D1
**Field**	IFL, IFM, IFH	IFN	IFN	IFN	IFN, unknown
**Hybrids**	37 (EA = 19, LA = 18)	Kelindos	Kelindos	Kelindos	Kelindos, unknown
**Fao class**	EA, LA	LA	LA	LA	LA, unknown
**Maturity**	EH, MH, LH	EH, MH, LH,VLH		EH, MH	
**Density**			DENl, DENh, none		

Method, fos = feed out simulating; inoculant, C = control, He = heterofermentative, Ho = homofermentative; dose, HD = half dose, SD = standard dose, DD double dose; delay, D0 = 0 h, D6 = 6 h, D20 = 24 h; input level field, IFL = low, IFM = medium, IFH = high, IFN = not estimated; Fao class, EA = early (FAO class = 200), LA = late (FAO class = 600–700); maturity, EH = 1/3 milk line, MH = 2/3 milk line phase, LH = 5 d after the 2/3 milk line, VLH = very late harvest (LH + 5 days); density, DENl = 140 kg DM/m^3^, DENh = 200 kg DM/m^3^.

### Methods

NIR instruments were used to analyse FHM and maize silage for proximate composition and fermentative profile. The NIR is used extensively in precision farming because it permits rapid, ecological and cheap on-farm analysis of forages [47–49] at cost-effectiveness in sample analysis [50]. Here, a NIR portable PoliSPEC^NIR^ (ITPhotonics srl, Breganze, Italy), together with robust calibration curves, was used to analyse DM, ash, CP, ether extract (**EE**), α-amylase NDF (**aNDF**), acid detergent fibre (**ADF**), sulfuric acid detergent lignin (lignin), WSC, and starch on FHM samples [12, 44]. Using the portable NIR prevents changes in FHM composition caused by respiration activity when analysis is delayed and enable a scan of a large proportion of the sample; therefore, the within-sample variability was acquired. A bench-top FOSS NIRSystem 5000 scanning monochromator (FOSS, Hillerød, Denmark) with the calibration described by Andrighetto et al. [11] was used to analyse silages. The reference methods utilised to calibrate the NIR instruments are described in Serva et al. [43].

The DM density (DMd, kg DM × m^-3^) was calculated as the amount of DM mass (kg of DM) filled in 1 m^3^ of volume (kg × m^-3^) [45]. The DM recovery (DMr, %) was calculated as the ratio of post-ensiled DMmass over the corresponding harvested pre-ensiled sample, while DM loss (%) was intended as 100-DMr. For Trials #2.a.1.a and #2.b, the post-ensiled DM mass was calculated as the sum of layers Spl, OT1, OT3, and OT5. The porosity was calculated according to the formula proposed by Richard et al. [43, 51]. The post-ensiled analysis results were utilised to calculate a fermentation quality index (FQI) according to what was reported by Andrighetto et al. for the quality index I1 [11]. The aerobic stability was defined as the time to exceed the room temperature of 2°C, while the event of instability was defined as the event of silage temperature that is 2°C over the room temperature [45].

The DM class at harvest was defined according to the DM content at harvest as low (< 30% DM), medium (30–35%), and high (> 35%).

### Statistical analysis

The statistic was performed using XLSTAT (XLSTAT, Addinsoft, release 2022.2.1, New York, USA) or R, version 4.0.2 (2020–06–22), which was used apart from Rcmdrpackage version 2.6–2 [52, 53], and RStudio Version 1.2.1578. The significance was declared at P ≤ 0.05, and trends at 0.05 < P < 0.10.

#### Exploratory data analysis

As an exploratory analysis and visual inspection of the dataset, a principal component analysis (PCA) was performed for continuous variables; the first two principal components (PC1 and PC2) were plotted, grouping samples for categorical variables (year, method, and inoculant). The Kaiser-Meyer-Olkin (KMO) test was used to evaluate the data set’s adequacy for the PCA. The value of KMO > 0.6 was considered adequate. A k-means algorithm was calculated on the PC and it revealed an adequate KMO and evaluated the grade of dissimilarity by the Euclidean distance and the agglomeration by using Ward’s method; the number of clusters was set between 2 and 10. The appropriate cluster number was selected according to the Silhouette index. Further, to find any association, the k-means-assigned class was matched with the qualitative variables of the dataset, and a confusion matrix and a contingency table were calculated. In a confusion matrix, metrics such as accuracy, the Matthew Correlation Coefficient (MCC), specificity, and sensitivity, were utilised. Moreover, the χ^2^ test calculated on the contingency table evaluated the association of the k-means-assigned class with the qualitative variables of the data set and the significance by cell (Fisher’s exact test).

All qualitative variables belonging to the data sets were tested for normality using the Shapiro–Wilk test (when W > 0.9, the data were considered normally distributed).

An ANCOVA that considered year, ensiling method, inoculant, dose, delay, DM class, input level field, FAO class, porosity, and density as fixed effects evaluated the chemical traits of FHM.

#### The effects of the pre-ensiling conditions on the silage’s fermentative traits

An ANCOVA that considered year, ensiling method, inoculant, dose, delay, DM class, input level field, FAO class, porosity and density as fixed effects evaluated the fermentative profile of silages, the DM loss and FQI. Moreover, the two-level interaction was also analysed. Post-hoc pairwise comparisons were run between factor levels using Bonferroni correction. Assumptions of the linear model on the residuals were graphically tested.

#### The effects of the pre-ensiling conditions on the silage’s aerobic stability

A Cox proportional hazard regression was separately tested for each covariate (univariable approach), and the event was referred to as aerobic instability. The proportional hazard assumption was evaluated using a visual approach (Schoenfeld, Martingala, beta, and score residuals) and Grambsh and Therneau test [54, 55].

#### Predictive models

Two predictive models were created. Initially, the pre-ensiling characteristics were employed to forecast the silage DM loss and FQI, utilizing a multivariable linear model (Lm) and a variable selection based on Akaike’s information criterion (Lm-AIC) in the backward direction.

Subsequently, the pre-ensiling traits were used to predict silage aerobic stability through a multivariable Cox model and forward/backward AIC (Cox-AIC) stepwise procedures. The Schoenfeld residuals were used to evaluate the proportional hazard assumption, while the variance inflation factor (VIF) evaluated the multicollinearity of predictors. In addition, Somer’s Dxy concordance index and R2 were used as metrics for the associations between predictors and outcomes.

The Lm-AIC and the Coc-AIC models were cross-validated across the years 2016 and 2022.

## Results

### Exploratory data analysis

**Fig 1:**
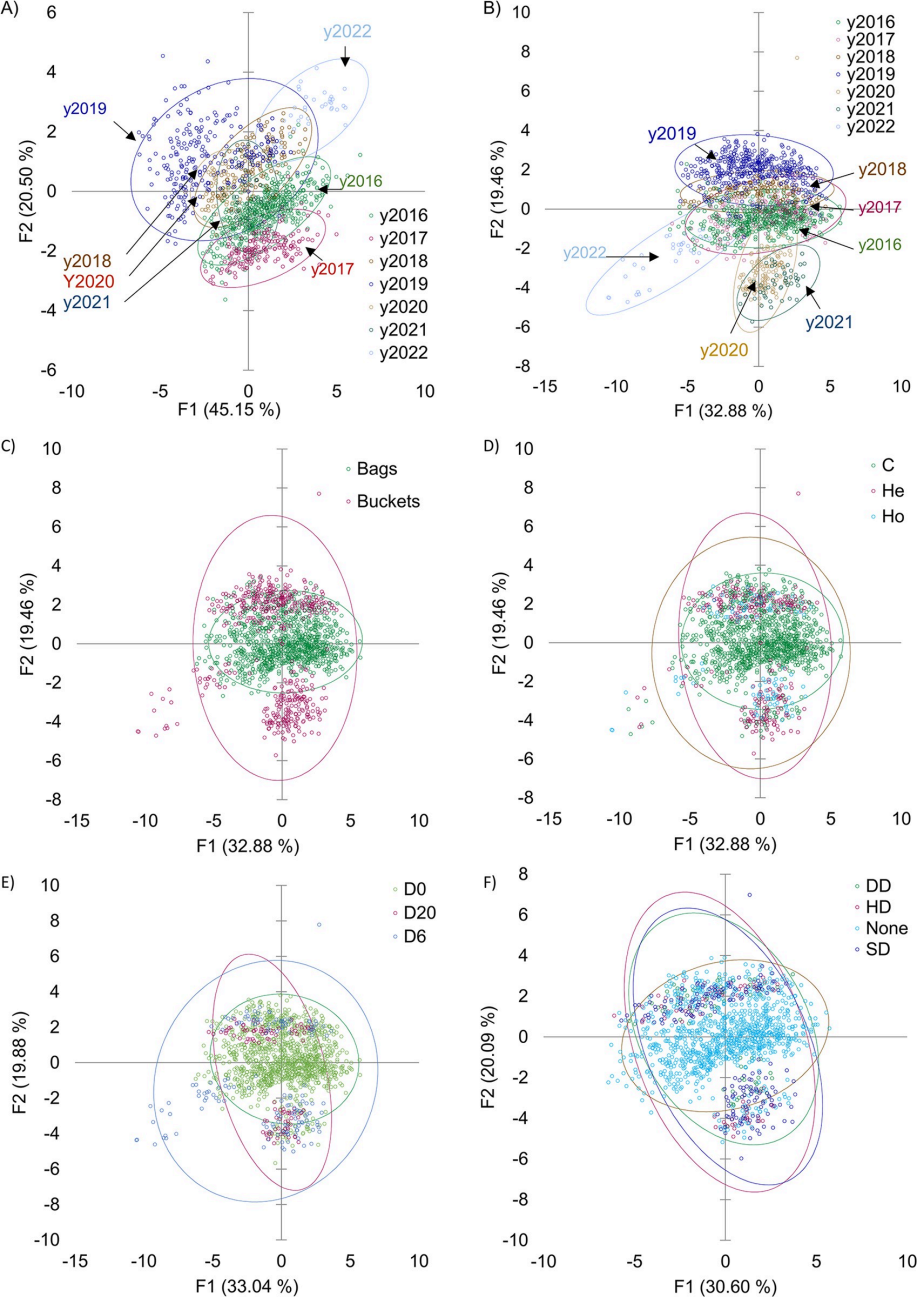
Scatter plot of the first two PC for the pre- and post-ensiled data set. Scatter plots displaying the first two principal components (PCs) for the pre-ensiled data set indicated by trial year (A), and for the post-ensile data set indicated by trial year (B), ensiling method (C), inoculant use (D), sealing delay (E), and different inoculant doses (F). The proportion of original variability explained by the first (F1) and second (F2) PCs is indicated in brackets. In (E) and (F), the percentage of explained variability differs due to missing values compared to (B), (C), and (D). Confidence intervals of 95% are represented by ovals. Legend: C = control; He = heterofermentative; Ho = homofermentative; D0 = delay 0 hours; D6 = delay 6 hours; D20 = delay 20 hours; HD = half dose; SD = standard dose; DD = double dose.

PCA reduces a database’s complexity, thereby transforming a large set of variables into a smaller set while retaining the essential information from the original data set. The principal components capture the most significant data variation sources, thereby allowing for simplified analysis and visualization while minimizing information loss. Here, the PCA was calculated in pre-ensiled traits, including proximate composition. The first two PCs captured over 65% of the original variability (9 PCs captured 99.9% of it), and the overall KMO was 0.60. Additionally, a PCA was calculated on post-ensiled traits, including proximate composition, fermentative profile, FQI and DM loss; in this manner, the PCA is projected to study differences related to the final silage quality. The first two PCs captured over 52% of the original variability (the 10 PCs captured 95.3%), and the overall KMO was 0.68. In the post-ensiled scatter plot, PC-2 increases in importance, and three clusters appear for the years 2016–2018, 2019, 2020–2021, and 2022 (Fig 1). In the case of the post-ensiled data set, for 2016, 2017, and 2018, they primarily overlap in samples ensiled with bags and without bacterial inoculation (C). This suggests that this particular ensiling method and treatment share similar characteristics in the data set for these three years. On the other hand, 2020 and 2021 appear grouped along PC2 and stand out from the other years of trials. This indicates that these two years exhibit distinct features compared to the remainder of the data set, but the reason for such segregation is unclear. As a tentative hypothesis, it can be inferred that meteorological conditions, even at a local level, may have influenced this segregation. In 2019, there is a partial separation along PC2, thereby suggesting that it possesses a few unique attributes or distinctions compared to other years. These observations imply the presence of year-specific trends or effects related to the ensiling method and inoculant usage, as revealed by the PCA analysis. Moreover, when using the buckets, two clusters emerge along PC2, approximately corresponding to the clusters of 2019, 2020, and Y2021.

The strength of the relationship between the PCs and initial variables can be assessed by examining the squared cosines of the variables (Table 2). These squared cosine values indicate the degree to which each original variable is associated with the respective PC. The highest squared cosine values, highlighted in bold, represent the primary variables that influence each PC.

**Table 2:** The squared cosine values for the original variables in the new features (PC1 to PC5) are provided below. Values in bold represent, for each variable, the factor for which the squared cosine is the largest.

	PC1	PC2	PC3	PC4	PC5
**Dry Matter (%)**	**0.843**	0.012	0.001	0.001	0.000
**Ash (% of DM)**	**0.482**	0.009	0.244	0.090	0.014
**Crude Protein (% of DM)**	0.294	0.021	0.047	**0.404**	0.018
**Ether Extract (% of DM)**	**0.352**	0.011	0.134	0.116	0.034
**aNDF (% of DM)**	**0.565**	0.128	0.032	0.139	0.002
**ADF (% of DM)**	**0.600**	0.198	0.015	0.083	0.004
**Starch (% of DM)**	**0.688**	0.082	0.009	0.092	0.031
**pH**	0.113	**0.474**	0.000	0.036	0.010
**Ammonia (Namm/Ntot, %)**	0.036	**0.539**	0.173	0.015	0.002
**Mannitol (% of DM)**	0.079	**0.687**	0.023	0.002	0.027
**Ethanol (% of DM)**	0.144	0.061	0.000	0.000	**0.687**
**Lactic ac. (% of DM)**	**0.574**	0.029	0.179	0.111	0.016
**Acetic ac. (% of DM)**	**0.367**	0.048	0.238	0.010	0.021
**Propionic ac. (% of DM)**	0.086	**0.429**	0.256	0.087	0.005
**Butyric ac. (% of DM)**	0.000	0.136	**0.516**	0.131	0.002
**DM loss (%)**	0.113	0.205	0.057	0.094	0.077
**FQI (Score 1–100)**	0.251	0.239	**0.264**	0.083	0.009

FQI = fermentation quality index; aNDF = α-amylase neutral detergent fibre; ADF = acid detergent fibre.

**Fig 2:**
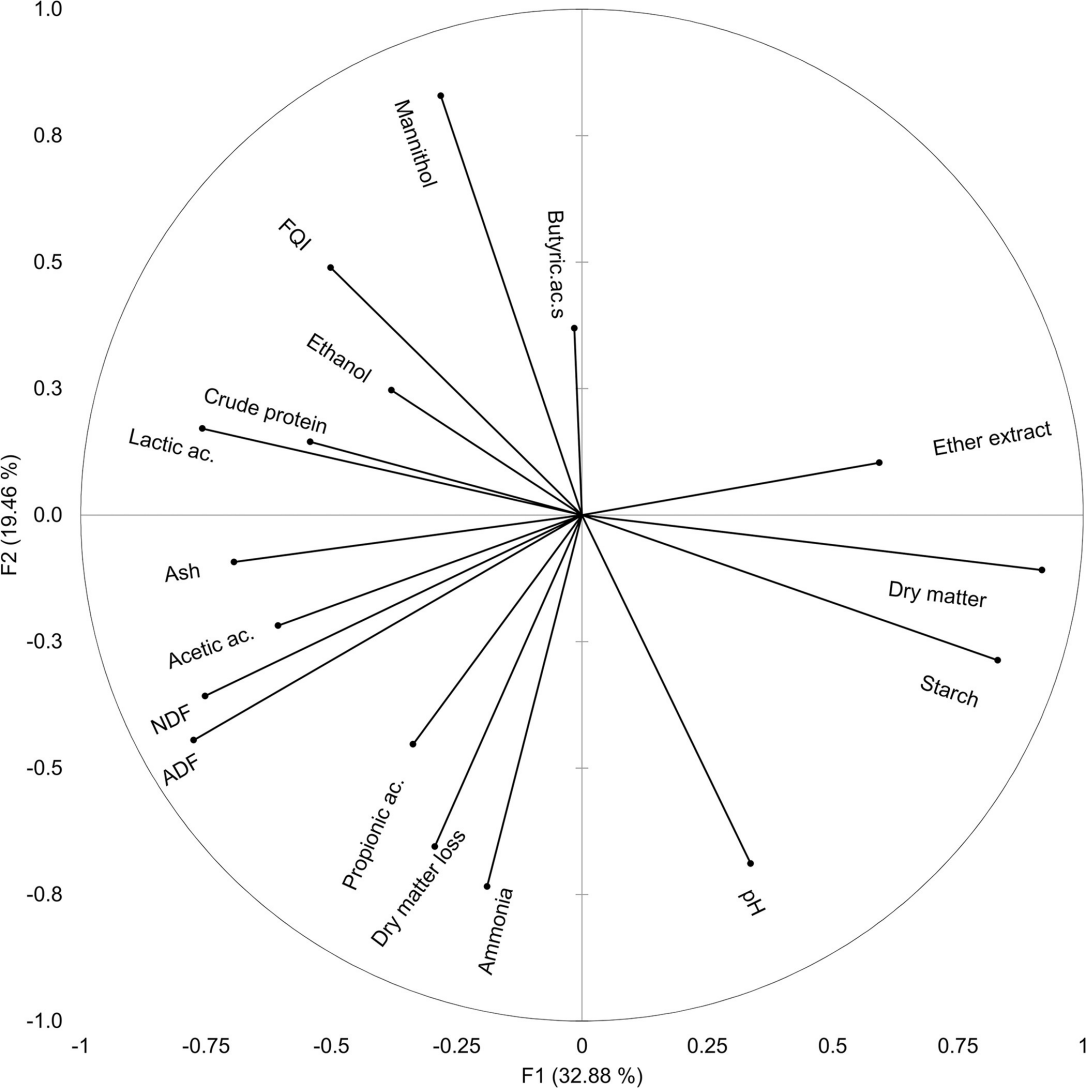
Contribution of the original post-ensiled variables to the principal components one (F1) and two (F2). The coordinate for each variable represents the correlation with PC1 and PC2. The proportion (%) of the original variability explained by PC1 (F1) and PC2 (F2) is reported in brackets. aNDF = α-amylase neutral detergent fibre; ADF = acid detergent fibre; FQI = fermentative quality index.

In Fig 2, the original variables are represented in the first two PCs, thererby illustrating their correlations with these two features. Upon visual inspection, it becomes apparent that DM and starch positively correlate with each other and PC1, but they exhibit a partial inverse relationship with lactic acid. Conversely, butyric acid reveals a weak positive correlation with PC2 and opposes with ammonia, DM loss and propionic acid. Since the two axes defined by PC1 and PC2 are independent, it is suggested that in maize silage, the ammonia, propionic acid, and butyric acid levels are unrelated to the content of DM, starch, and, to a certain extent, lactic acid.

The k-means analysis applied to PC 1–5 yielded an optimal number of six clusters, corresponding to a Silhouette index of 0.331, thererby indicating that the data was most effectively grouped into six clusters based on the selected features or principal components. Meanwhile, the within-cluster sum of squares (inertia) decreases until at least ten classes, thereby suggesting that more clusters can be appropriately utilised. However, it’s worth noting that the data set lacks a qualitative variable with six levels to directly compare with the six k-means assigned clusters. Therefore, the two methods, three inoculant types, three sealing delays, four inoculant doses, and seven years were compared with two, three, three, four, and seven k-means assigned clusters (Table 3).

**Table 3:** The confusion matrix was computed separately for two, three, four, and seven clusters from k-means clusters related to the ensiling method inoculant, sealing delay, inoculant dose, and years, respectively. In parentheses, it is indicated whether the observed value is lower (<) or higher (>) than the expected value, along with the significance from Fisher’s exact test (Χ^2^ test).

cluster	bags	buckets						sum
**1**	762 (>, ****)	275 (<, ****)						1037
**2**	240 (<, ****)	219 (>, ****)						459
**sum**	1002	494						1496
**χ**^**2**^ **tets**	P < 0.0001							
**sensitivity**	0.76	0.44						
**specificity**	0.44	0.76						
**accuracy**	0.66	0.66						
**precision**	0.73	0.48						
**MCC**	0.21	0.21						
**cluster**	C	He	Ho					sum
**1**	540 (>, ****)	66 (<, ****)	20 (<, ****)					626
**2**	535 (>, ****)	93 (<, ****)	32 (<, ****)					660
**3**	57 (<, ****)	102 (>, ****)	51 (>, ****)					210
**sum**	1132	261	103					1496
**χ**^**2**^ **tets**	P < 0.0001							
**sensitivity**	0.48	0.36	0.50					
**specificity**	0.76	0.54	0.89					
**accuracy**	0.55	0.51	0.86					
**precision**	0.86	0.14	0.24					
**MCC**	0.21	-0.08	0.28					
**cluster**	D0	D6	D20					sum
**1**	**566** (>, ****)	28 (<, ****)	32 (<, **)					626
**2**	592 (>, ****)	**39** (<, ****)	29 (<, ***)					660
**3**	66 (<, ****)	98 (>, ****)	**46** (>, ****)					210
**sum**	1224	165	107					1496
**χ**^**2**^ **tets**	P < 0.0001							
**sensitivity**	0.46	0.24	0.43					
**specificity**	0.78	0.53	0.88					
**accuracy**	0.52	0.50	0.85					
**precision**	0.90	0.06	0.22					
**MCC**	0.19	-0.15	0.23					
	none	HD	SD	DD				sum
**1**	528 (>, ****)	1 (<, ****)	14 (<, ****)	8 (<, ****)				551
**2**	279 (>, ****)	7 (<, ***)	47 (<)	3 (<, ****)				336
**3**	45	27 (>, ****)	83 (>, ****)	21 (>, ****)				176
**4**	268	40 (>, ****)	82 (>, **)	43 (>, ****)				433
**sum**	1120	75	226	75				1496
**χ**^**2**^ **tets**	P < 0.0001							
**sensitivity**	0.24	0.05	0.18	0.29				
**specificity**	0.97	0.88	0.96	0.86				
**accuracy**	0.42	0.84	0.85	0.83				
**precision**	0.96	0.02	0.47	0.10				
**MCC**	0.23	-0.05	0.22	0.09				
	y2016	y2017	y2018	y2019	y2020	y2021	y2022	sum
**1**	144 (>, ****)	75 (>, ****)	8 (<, ***)	2 (<, ****)	4 (<, ****)	0 (<, ***)	0 (<, **)	233
**2**	192 (>, ****)	47 (>, ***)	6 (<, ****)	4 (<, ****)	0 (<, ****)	0 (<, ***)	1 (<, *)	250
**3**	16 (<, ****)	8 (<)	**15 (>)**	35 (>)	0 (<, ****)	0 (<, *)	35 (>, ****)	109
**4**	170 (>, ****)	49 (>, ****)	0 (<, ****)	2 (<, ****)	5 (<, ****)	1 (<, ***)	0 (<, ***)	227
**5**	0 (<, ****)	1 (<, ****)	0 (<, ****)	0 (<, ****)	125 (>, ****)	47 (>, ****)	0 (<, *)	173
**6**	0 (<, ****)	0 (<, ****)	41 (>, **)	258 (<, ****)	0 (<, ****)	0 (<, ****)	0 (<, ***)	299
**7**	0 (<, ****)	0 (<, ****)	74 (>, ****)	130 (>, ****)	1 (<, ****)	0 (<, ***)	0 (<, *)	205
**sum**	522	180	144	431 (>, ****)	135 (<, ****)	48	36	1496
**χ**^**2**^ **tets**	P < 0.0001							
**sensitivity**	0.28	0.26	0.10	0.00	0.93	0.00	0.00	
**specificity**	0.91	0.85	0.93	0.79	0.96	0.80	0.86	
**accuracy**	0.69	0.78	0.85	0.56	0.96	0.78	0.84	
**precision**	0.62	0.19	0.14	0.01	0.72	0.00	0.00	
**MCC**	0.24	0.09	0.04	-0.26	0.80	-0.09	-0.06	

C = control; He = heterofermentative; Ho = homofermentative; D0 = delay 0 h; D6 = delay 6 h; D20 = delay 20 h; HD = half dose; SD = standard dose; DD = double dose. Significance P = 0.05 (*); P = 0.01 (**); P = 0.001 (***); P < 0.0001 (****).

**Fig 3:**
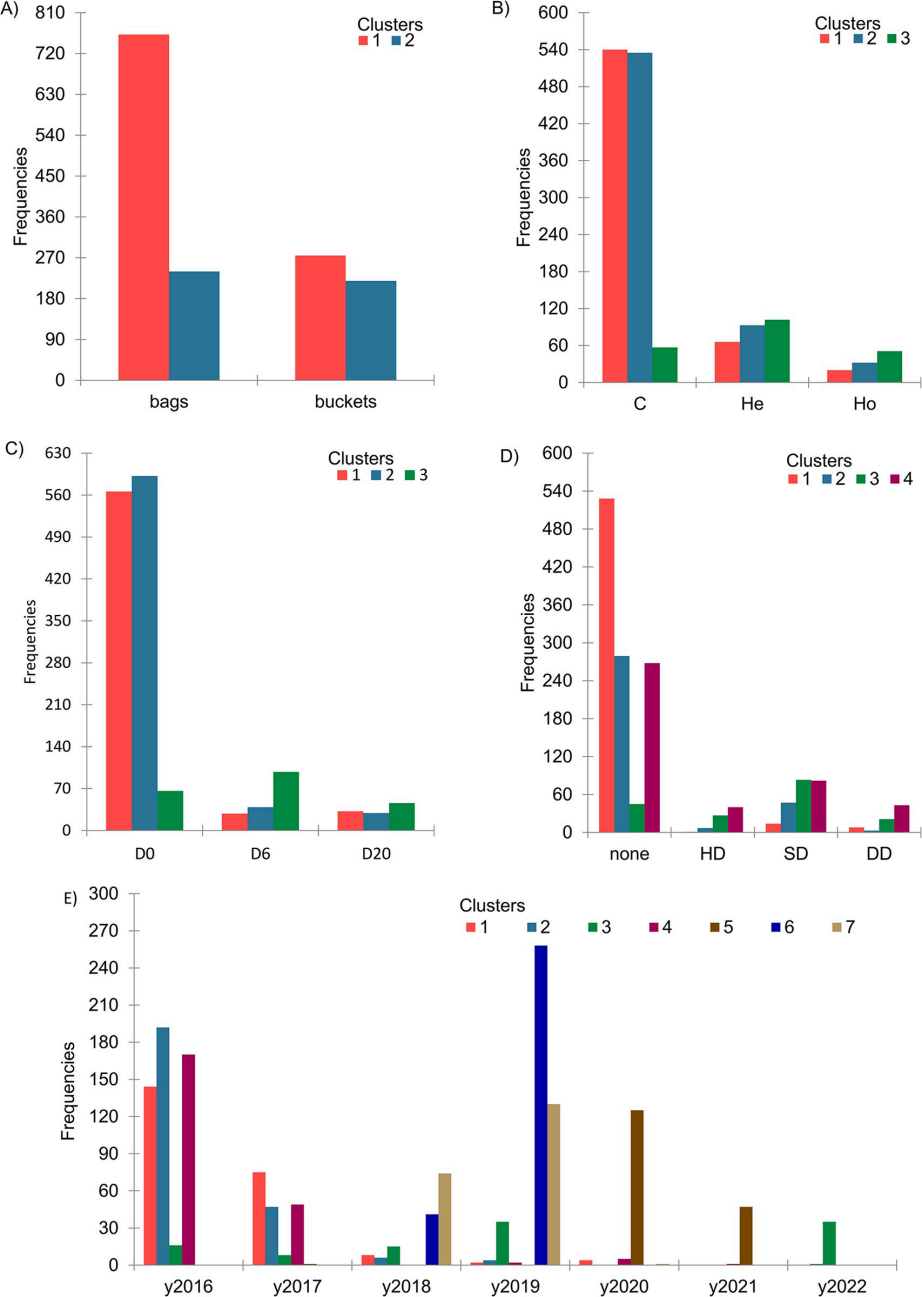
The frequency distribution for assigned k-means clusters. The assigned frequency distribution of two, three, four, and seven clusters is presented for the following factors: ensiling method (A), inoculant (B), ensuing delay (C), inoculant dose (D), and years (E).). C = control; He = heterofermentative; Ho = homofermentative; D0 = delay 0 h; D6 = delay 6 h; D20 = delay 20 h; HD = half dose; SD = standard dose; DD = double dose.

Although the χ2 tests conducted for the two, three, four and seven clusters, suggest a connection between clusters and variables, the results from the confusion matrix are less straightforward to interpret. As demonstrated in the visual representation of Fig 3 and Table 3, the relationship between clusters and variables remains unclear and challenging to decipher.

The first two PC scatter plots for the two, three, four and seven k-means assigned classes are reported in S1 Fig. Upon comparing these scatter plots with those in Fig 1, it is evident that the methods do not intersect cluster two, the inoculants do not coincide with cluster three, and the years (from pre- or post-ensiled data sets) do not flawlessly correspond to cluster seven as assigned by k-means. However, in the post-ensiled data set, 2020 and 2021 cluster together and are aligned with cluster #5, whereas 2020 partially aligns with cluster #3, 2019 overlaps clusters #6 and #7, and 2017 together with 2018 coincides with clusters #1, #2, and #4 from the k-means assigned cluster seven. Generally, based on the information presented in S1 Fig, the k-means assigned clusters two to seven appear to provide additional perceptions related to the years of the trial.

Upon closer examination of the figures, it becomes evident that when examining at the histograms (Fig 3A and S1A Fig), the method bags primarily align with cluster #1 and, to a certain extent, with cluster #2. The latter portion of cluster #2 primarily pertains to 2020 and 2021, as indicated by Fig 1A and 1C.

Similarly, when examining the figures related to the use of inoculants, clusters #1 and #2 primarily comprise control samples and those treated with He and Ho from 2019, as indicated in Figs 1B and 3B, and S1B Fig). Similar observations can be made regarding the sealing delay, mirroring those discussed in the context of inoculant usage. Finally, in the case of the dose, cluster #4 mostly fits with the SD belonging to 2020 + 2021, and cluster #4 matches with 2019.

Further, an ANCOVA considering year, ensiling method, inoculant, dose, delay, DM class, input level field, FAO class, porosity, and density as fixed effects was utilised to evaluate the FHM traits (Table 4).

**Table 4:** The ANCOVA was performed, considering the year, ensiling method, inoculant, dose, delay, DM class, input level field, and FAO class as fixed effects and assessing the chemical composition of freshly harvested maize (FHM). If not specified, values are expressed as % of DM.

Pre-ensiled factors	DM (%)	Ash	CP	EE	aNDF	ADF	Lignin	WSC	Starch
**y2016**	35.3 b	4.15 b	6.78 c	2.23 e	46.9 a	24.8 a	2.50 b	4.66 e	24.5 e
**y2017**	35.3 b	4.13 b	6.59 d	1.83 f	44.8 b	23.4 b	1.81 d	5.31 d	20.6 f
**y2018**	35.1 b	3.76 c	6.56 d	2.62 d	44.7 b	24.7 a	2.87 a	6.27 c	28.2 d
**y2019**	35.0 b	3.33 e	6.76 c	3.13 a	37.2 d	19.8 d	2.25 c	7.56 b	35.1 a
**y2020**	35.3 b	3.47 d	6.34 e	3.02 b	36.2 e	19.6 d	2.22 c	7.32 b	33.3 b
**y2021**	38.9 a	4.06 b	7.37 b	2.88 c	40.3 c	21.0 c	1.89 d	6.64 c	31.0 c
**y2022**	32.3 c	4.78 a	8.99 a	2.26 e	39.3 c	20.3 cd	1.81 d	10.7 a	30.4 c
**SEM**	0.31	0.03	0.04	0.03	0.31	0.21	0.03	0.14	0.37
**P**	<0.0001	<0.0001	<0.0001	<0.0001	<0.0001	<0.0001	<0.0001	<0.0001	<0.0001
**DM class low**	31.1 c	4.09 a	7.10 a	2.47 c	42.7 a	23.5 a	2.37 a	6.80 b	27.4 c
**DM class medium**	34.4 b	3.98 b	7.03 b	2.53 b	41.6 b	22.0 b	2.21 b	7.06 a	28.3 b
**DM class high**	40.4 a	3.79 c	7.04 ab	2.70 a	39.7 c	20.3 c	2.00 c	6.93 ab	31.3 a
**SEM**	0.22	0.02	0.028	0.02	0.228	0.15	0.019	0.10	0.26
**P**	<0.0001	<0.0001	<0.0001	<0.0001	<0.0001	<0.0001	<0.0001	<0.0001	<0.0001
**IFN**	36.5 a	3.96 b	7.15 a	2.66 a	40.2 b	21.1 b	2.10 b	7.13 a	29.7 a
**IFH**	34.3 b	3.88 c	7.15 a	2.67 a	39.7 b	20.9 bc	2.07 bc	7.16 a	30.0 a
**IFM**	35.9 a	4.19 a	6.89 c	2.29 b	45.8 a	25.0 a	2.56 a	6.16 b	26.2 b
**IFL**	34.7 b	3.79 d	7.04 b	2.66 a	39.6 b	20.6 c	2.04 c	7.26 a	30.2 a
**SEM**	0.25	0.03	0.032	0.022	0.26	0.17	0.02	0.11 a	0.30 a
**P**	<0.0001	<0.0001	<0.0001	<0.0001	<0.0001	<0.0001	<0.0001	<0.0001	<0.0001
**EH**	33.0 d	3.93 b	6.99 b	2.56 bc	40.5 c	21.568 bc	2.11 b	7.39 a	29.1 b
**MH**	33.7 c	3.87 b	7.09 a	2.65 a	40.4 c	21.517 c	2.18 a	6.91 b	30.2 a
**LH**	36.1 b	3.86 b	6.98 b	2.60 b	41.3 b	21.962 ab	2.20 a	6.22 c	29.6 ab
**VLH**	38.5 a	4.16 a	7.16 a	2.46 c	43.1 a	22.669 a	2.27 a	7.19 ab	27.0 c
**SEM**	0.26	0.03 b	0.03	0.02	0.26	0.171	0.02 a	0.11	0.30
**P**	<0.0001	<0.0001	<0.0001	<0.0001	<0.0001	<0.0001	<0.0001	<0.0001	<0.0001
**EA**	35.5	3.88	6.99	2.57	41.3	21.8	2.14	6.93	29.1
**LA**	35.1	4.02	7.12	2.56	41.4	22.1	2.24	6.93	28.9
**SEM**	0.21	0.02	0.03	0.02	0.21	0.14	0.02	0.09	0.24
**P**	<0.0001	<0.0001	<0.0001	<0.0001	<0.0001	<0.0001	<0.0001	<0.0001	<0.0001

DM = dry matter; CP = crude protein; EE = ether extract; WSC = water-soluble carbohydrate; aNDF = alfa-amylase neutral detergent fibre; ADF = acid detergent fibre; IFN = Input field not estimated; IFH = input field high; IFM = input field medium; IFL = input field low; EH = early harvesting; MH = medium harvesting; LH = late harvesting; VLH = very late harvesting; EA = FAO class early; LA = FAO class late.

As expected, the factor year affected all the FHM chemical traits. Interestingly, the FHM from 2016 to 2020 exhibited similar DM at harvest, while the other traits varied across those years. However, the chemical traits generally appear to differ, on average, in the DM class at harvest (increasing or decreasing with DM), except for WSC, which is an unclear trend.

The input field, the maturity at harvest, and the FAO class influenced all FHM traits. The IFH samples, compared to IFL, have similar DM at harvest, but higher CP and EE and similar fibre fractions, WSC, and sugars. Further, as expected, the level of maturity at harvest was directly related to the DM content in FHM. In addition, starch content increased from early maturity to late maturity but decreased from late maturity to very late maturity, while the WSC content exhibited the opposite trend. This variation can be attributed to increased NDF content from early to very late maturity, which dilutes the other traits. With regards to the contrast between early and late FAO class hybrids, all traits reveal statistical but not functional differences between EA and LA. However, it becomes evident that this data set does not exhibit a consistent linear regression for WSC by the DM (S2 Fig).

### The effects of the pre-ensiling conditions on the silage’s fermentative traits

An ANCOVA that considered year, ensiling method, inoculant, dose, delay, DM class, input level field, FAO class, porosity, and density as fixed effects was performed to evaluate the chemical traits of silage (Table 5).

**Table 5:** The ANCOVA was performed, considering year, ensiling method, inoculant, dose, delay, DM class, input level field, FAO class, porosity, and density as fixed effects, and assessed the fermentative profile of silages, DM loss, and FQI. Additionally, two-level interactions were examined.

Pre-ensiled factors	pH	Ammonia^1^	Mannitol^2^	Ethanol^2^	Lactic ac. ^2^	Acetic ac. ^2^	Propionic ac. ^2^	Butyric ac. ^2^	DM loss^3^	FQI^4^
**2016**	3.86 c	5.55 c	1.27 c	0.74 b	5.43 b	0.84 e	0.02 d	0.05 cd	11.7c	58.9 ab
**2017**	3.87 c	5.42 d	1.33 c	0.23 d	5.25 c	1.19 d	0.10 c	0.04 d	10.4 c	61.4 a
**2018**	3.74 e	4.47 e	2.09 a	0.42 c	3.88 e	1.54 c	0.01 d	0.15 a	14.07 b	47.5 c
**2019**	3.79 d	3.91 f	1.84 b	0.98 a	4.46 d	1.11 d	0.01 d	0.12 b	11.0 c	57.3 b
**2022**	3.88 c	6.96 a	0.78 d	1.26 a	6.82 a	2.25 a	1.06 a	0.13 ab	14.8 b	48.5 c
**2020**	3.95 b	5.93 b	0.19 e	0.41 cd	3.77 e	1.59 c	0.67 b	0.05 cd	18.8 a	43.6 d
**2021**	4.03 a	6.60 a	0.28 e	0.29 cd	2.90 f	1.80 b	0.68 b	0.06 c	13.9 b	28.9 e
**SEM**	0.02	0.11	0.08	0.10	0.15	0.07	0.02	0.01	1.12	1.60
**P**	<0.0001	<0.0001	<0.0001	<0.0001	<0.0001	<0.0001	<0.0001	<0.0001	<0.0001	<0.0001
**Bags**	3.86	5.85	0.86	1.35	4.83	1.77	0.49	0.10	12.3	46.3
**Buckets**	3.89	5.25	1.36	0.12	4.46	1.18	0.21	0.08	14.7	52.5
**SEM**	0.03	0.18	0.12	0.15	0.22	0.11	0.03	0.01	1.65	2.36
**P**	0.647	0.072	0.029	<0.0001	0.374	0.004	<0.0001	0.355	0.438	0.165
**C**	3.89	5.21 b	1.22 a	0.55	3.93 b	1.55 a	0.38 a	0.08 a	13.1	48.2
**He**	3.86	5.84 a	1.01 a	0.59	5.01 a	1.53 a	0.37 a	0.09 a	13.5	49.9
**Ho**	3.87	5.59 b	1.11 a	0.72	5.01 a	1.36 b	0.30 b	0.09 a	13.9	50.2
**SEM**	0.02	0.14	0.09	0.12	0.17	0.08	0.02	0.01	1.27	1.81
**P**	0.619	<0.0001	0.039	0.071	<0.0001	<0.0001	<0.0001	0.250	0.754	0.713
**none**	3.86	6.05 a	0.98	0.68	5.41 a	1.47 ab	0.34 ab	0.10	14.4	49.0 b
**HD**	3.90	5.50 b	1.16	0.61	4.33 b	1.60 a	0.38 a	0.09	12.9	47.5 b
**SD**	3.87	5.46 b	1.09	0.60	4.38 b	1.55 a	0.39 a	0.08	13.2	49.3 b
**DD**	3.87	5.19 c	1.21	0.60	4.48 b	1.30 b	0.30 b	0.08	13.5	52.0 a
**SEM**	0.02	0.14	0.10	0.12	0.17	0.08	0.02	0.01	1.27	1.82
**P**	0.081	<0.0001	0.086	0.971	0.0003	<0.0001	<0.0001	0.181	0.753	<0.0001
**D0**	3.87	5.58	1.11	0.63	4.57	1.41 b	0.34 b	0.09	10.2 c	48.4
**D6**	3.86	5.50	1.12	0.56	4.64	1.43 b	0.34 b	0.08	12.7 b	49.5
**D20**	3.89	5.57	1.09	0.67	4.73	1.59 a	0.37 a	0.09	17.7 a	50.4
**SEM**	0.02	0.11	0.08	0.09	0.14	0.07	0.02	0.01	1.01	1.44
**P**	0.070	0.323	0.827	0.167	0.117	<0.0001	0.010	0.352	<0.0001	0.057
**DM class low**	3.86	5.67 a	1.31 a	0.71 a	5.22 a	1.70 a	0.39 a	0.07 b	14.0 a	53.2 a
**DM class medium**	3.88	5.60 a	1.08 b	0.53 b	4.58 b	1.56 b	0.38 a	0.09 a	12.4 b	49.2 b
**DM class high**	3.89	5.37 b	0.95 c	0.62 ab	4.15 c	1.18 c	0.28 b	0.10 a	14.1 a	45.9 c
**SEM**	0.02	0.11	0.09	0.09	0.14	0.07	0.02	0.01	1.00	1.43
**P**	0.087	<0.0001	<0.0001	0.000	<0.0001	<0.0001	<0.0001	<0.0001	0.000	<0.0001
**IFN**	3.86 c	5.53 b	1.19 a	1.66 a	5.27 a	1.78 a	0.54 a	0.11	9.68 c	46.2 c
**IFH**	3.88 b	5.53 b	1.17 a	0.08 c	4.55 a	1.36 b	0.29 c	0.08	13.8 b	54.0 a
**IFM**	3.86 c	5.70 a	0.98 c	0.57 b	4.56 a	1.41 b	0.27 c	0.08	16.1 a	49.3 b
**IFL**	3.91 a	5.43 c	1.10 b	0.16 c	4.21 b	1.36 b	0.31 b	0.08	14.4 b	48.3 b
**SEM**	0.03	0.16	0.08	0.14	0.21	0.10	0.02	0.01	1.54	2.20
**P**	<0.0001	<0.0001	<0.0001	<0.0001	<0.0001	0.017	<0.0001	0.102	<0.0001	<0.0001
**EH**	3.86	5.65 a	1.04 b	0.76 a	5.01 a	1.54 a	0.35 b	0.08 b	14.3 a	53.8 a
**MH**	3.87	5.37 b	1.21 a	0.50 c	4.78 b	1.45 b	0.33 c	0.07 b	13.2 ab	53.0 a
**LH**	3.88	5.47 b	1.17 a	0.65 ab	4.63 c	1.41 b	0.31 d	0.07 b	13.7 ab	49.9 b
**VLH**	3.88	5.71 a	1.03 b	0.56 bc	4.16 d	1.52 ab	0.42 a	0.13 a	12.8 b	41.1 c
**SEM**	0.02	0.11	0.08	0.10	0.14	0.07	0.02	0.03	1.00	1.48
**P**	0.063	<0.0001	<0.0001	<0.0001	<0.0001	<0.0001	<0.0001	<0.0001	0.006	<0.0001
** **	pH	Ammonia	Mannitol	Ethanol	Lactic ac.	Acetic ac.	Propionic ac.	Butyric ac.	DM loss	FQI
**EA**	3.89	5.52	1.23	0.64	4.87 a	1.48	0.34 b	0.09	13.8	50.4
**LA**	3.86	5.58	0.99	0.60	4.42 b	1.47	0.36 a	0.09	13.3	48.4
**SEM**	0.02	0.10	0.07	0.09	0.13	0.06	0.02	0.01	0.96	1.36
**P**	<0.0001	0.071	<0.0001	0.147	<0.0001	0.522	0.006	0.231	0.077	<0.0001
**C×D0**	3.88	5.17 e	1.24	0.56	3.67 c	1.45	0.37	0.094 a	8.27 c	45.7
**He×D0**	3.87	5.97 e	0.99	0.57	5.10 a	1.48	0.37	0.086 ab	10.7 bc	50.0
**Ho×D0**	3.88	5.60 e	1.11	0.75	4.94 ab	1.31	0.29	0.090 ab	11.6 b	49.5
**C×D6**	3.89	5.28 e	1.19	0.51	4.04 c	1.54	0.38	0.075 b	12.8 b	48.9
**He×D6**	3.84	5.70 e	1.06	0.52	4.93 ab	1.48	0.36	0.088 ab	13.3 b	50.1
**Ho×D6**	3.86	5.52 e	1.12	0.66	4.96 ab	1.28	0.28	0.093 a	11.9 b	49.59
**C×D20**	3.90	5.19 e	1.23	0.58	4.07 bc	1.66	0.41	0.082 ab	18.3 a	49.9
**He×D20**	3.88	5.9 abc	0.96	0.68	5.00 a	1.64	0.39	0.088 ab	16.5 a	49.7
**Ho×D20**	3.88	5.7 abc	1.09	0.74	5.13 a	1.48	0.33	0.095 a	18.2 a	51.6
**P**	0.583	0.026	0.621	0.898	0.002	0.711	0.736	0.018	0.001	0.057
**C × DM class low**	3.86	5.46 de	1.40	0.55 bc	4.64 cd	1.74 a	0.40 ab	0.08 de	14.0abc	52.3 ab
**He × DM class low**	3.85	5.90 ab	1.26	0.68 ab	5.67 a	1.73 ab	0.41 a	0.06 f	13.8 abc	54.8 a
**Ho × DM class low**	3.87	5.66 abcd	1.26	0.90 a	5.34 b	1.62 abc	0.38 ab	0.07 ef	14.1 ab	52.6 ab
**C × DM class medium**	3.89	5.25 e	1.21	0.57 bc	3.91 e	1.57 bc	0.38 ab	0.09 cde	12.811 abc	48.7 cd
**He×DM class medium**	3.87	5.90 a	0.92	0.45 c	4.93 c	1.62 abc	0.40 a	0.10 abc	11.7 c	49.3 bc
**Ho × DM class medium**	3.88	5.65 bcd	1.10	0.56 bc	4.92 c	1.50 c	0.35 b	0.10 ab	12.7 abc	49.6 bc
**C × DM class high**	3.92	4.93 f	1.05	0.53 bc	3.23 f	1.33 d	0.37 ab	0.09 bcd	12.5 bc	43.5 e
**He × DM class high**	3.88	5.72 abc	0.83	0.64 bc	4.45 d	1.24 d	0.30 c	0.10 a	15.0 a	45.7 de
**Ho × DM class high**	3.87	5.48 cde	0.96	0.69 ab	4.76 cd	0.95 e	0.17 d	0.11 a	14.9 ab	48.4 cd
**P**	0.093	0.003	0.130	0.0001	<0.0001	0.0004	<0.0001	<0.0001	0.0002	0.016

1 = (Namm/Ntot, %); 2 = % of the DM; 3 = %; 4 = scores 1–100; DM = dry matter; C = control; He = heterofermentative; Ho = homofermentative; D0 = delay 0 h; D6 = delay 6 h; D20 = delay 20 h; none = non-inoculants; HD = half dose; SD = standard dose; DD = double dose; IFN = Input field not estimated; IFH = input field high; IFM = input field medium; IFL = input field low; EH = early harvesting; MH = medium harvesting; LH = late harvesting; VLH = very late harvesting; EA = FAO class early; LA = FAO class late.

Based on the data outlined in Tables 4 and 5, it is evident that the year of the trial had a significant impact on all the examined parameters. Specifically, 2021 exhibited the highest DM at harvest and silage pH, whereas 2022 revealed the lowest DM at harvest, the lowest pH, and the highest VFA content in the silage. The choice of the ensiling method (bags or buckets) affected the acetic and propionic acid levels but not the lactic acid and the pH levels. Further, inoculant usage (He or Ho) increased the lactic acid content, while the acetic acid content was lower in Ho compared to He and the control. The dose of inoculant partially impacted VFA, with the double dose not indicating significant improvements in lactic or acetic acid content compared to the standard or half dose.

Low IFLs reduced lactic acid and increased pH compared to medium (IFM) or high (IFH) IFLs. Additionally, earlier harvests were generally linked to higher VFA content, FQI, and DM loss. However, no linear trend existed between DM at harvest and DM loss or FQI. Lastly, the use of inoculants (He and Ho) led to higher lactic acid content in delayed sealed silages and in maize harvested with higher DM, that in the control group.

### The effects of the pre-ensiling conditions on silage’s aerobic stability

A Cox univariable model helps identify the factors that may protect or predispose silage to aerobic instability (measured by survival time), as presented in Table 6.

**Table 6:** Univariable Cox model hazard ratio (HR) for the pre-ensiling traits and ensiling conditions; the distribution of samples undergoing different ensiling conditions and the average composition for freshly harvested maize for all samples, stable and unstable samples.

Cox Model Univariable					
Variable	All sample	Stable	Unstable	HR	HR, 95% C.I.
Distribution of samples, N (%)					
**Year (y2018)**	16 (10.0)	15 (93.8)	1 (6.25)		
**2019**	36 (22.5)	19 (52.8)	17 (47.2)	9.32	1.24–70.1
**2020**	36 (22.5)	24 (66.7)	12 (33.3)	5.77	0.75–44.4
**2021**	36 (22.5)	8 (22.2)	28 (77.8)	5.11	0.67–39.0
**2022**	36 (22.5)	0 (0)	36 (100)	33.3	4.53–245
**Inoculant (Control)**	48 (30.0)	16 (33.3)	32 (66.7)		
**He**	86 (53.8)	50 (58.1)	36 (41.9)	0.47	0.29–0.80
**Ho**	26 (16.3)	0 (0)	26 (100)	1.15	0.67–2.00
**Dose (none)**	31 (29.2)	16 (51.6)	15 (48.4)		
**HD**	18 (17.0)	9 (50.0)	9 (50)	1.24	0.53–2.86
**SD**	39 (36.8)	25 (64.1)	14 (35.9)	0.40	0.18–0.89
**DD**	18 (17.0)	12 (66.7)	6 (33.3)	0.82	0.31–2.13
**Delay (D0)**	59 (36.9)	35 (59.3)	24 (40.7)		
**D6**	77 (48.1)	17 (22.1)	60 (77.9)	2.21	1.37–3.57
**D20**	24 (15.0)	14 (58.3)	10 (41.7)	1.47	0.70–3.10
**Maturity (EH)**	36 (40.0)	29 (80.6)	7 (19.4)		
**MH**	12 (13.3)	0 (0)	12 (100)	7.39	2.87–19
**LH**	32 (35.6)	14 (43.8)	18 (56.3)	2.69	1.09–6.66
**VLH**	10 (11.1)	4 (40)	6 (60.0)	0.59	0.16–2.23
Average (standard deviation)					
**DM**	33.5 (6.50)	34.9 (6.5)	32.5 (6.32)	0.89	0.86–0.93
**Ash**	4.20 (0.63)	3.80 (0.31)	4.48 (0.64)	1.13	0.18–6.39
**CP**	7.18 (1.06)	6.51 (0.44)	7.66 (1.11)	2.13	1.73–2.62
**EE**	2.53 (0.42)	2.76 (0.23)	2.37 (0.44)	0.25	0.16–0.39
**aNDF**	42.9 (2.93)	42.6 (3.52)	43.0 (2.39)	0.98	0.91–1.06
**ADF**	23.7 (2.11)	23.9 (2.5)	23.6 (1.77)	1.02	0.92–1.13
**Lignin**	2.49 (0.39)	2.66 (0.4)	2.37 (0.33)	0.94	0.52–1.7
**WSC**	7.43 (2.00)	6.20 (1.14)	8.32 (2.02)	1.45	1.32–1.6
**Starch**	29.4 (3.43)	30.5 (3.74)	28.6 (2.9)	0.89	0.83–0.96
**Porosity**	0.58 (0.08)	0.56 (0.08)	0.59 (0.07)	0.01	0–0.25
**Density**	172 (29.5)	183 (24)	164 (31)	0.98	0.98–0.99

Categorical variables are presented as the number of samples and their percentage (in parentheses) within each factor considered (years., inoculant, dose, delay, and maturity class). The effect of the year (from 2018 to 2022), the use of inoculants (C = control; He = heterofermentative; Ho = Homofermentative), the dose of inoculant (none, HD = half dose; SD = standard dose; DD = double dose), the harvesting maturity stage (EH = early harvested; MH = medium harvested; LH = late harvested; VHL = very late harvest) and the ensiling delays time (D0 = 0h, D6 = 6h, D20 = 20h) are presented. The univariate hazard ratio (HR) and 95% C.I. are reported for individual variables for the event of aerobic-unstable silage. HR refers to 2018 for years of harvesting, to C for the use of inoculants, to none for the dose of inoculant, to EH for the harvesting maturity stage, and to D0 for the ensiling delay time.

The years 2019 and 2022, both of which exhibited DM at a harvest level lower than 35%, were found to be predisponents to aerobic instability compared to 2018. Notably, 2022 demonstrated HR = 33.3 and was characterised by a lower pH value, a higher VFA value, and increased DM loss, while maintaining a medium FQI value.

Consistent with expectations, heterofermentative inoculants proved to be protective compared to the control, as did utilizing the standard dose compared to the control group without any dose. With regards to the sealing delay, D6 emerged as a predisposing factor for aerobic instability, while D20 did not when compared to a fast silo sealing (D0). Finally, increased plant maturity at harvest was associated with a higher likelihood of aerobic instability when compared to early harvest (EH).

Further, among the FHM traits, DM, EE, and starch were identified as protective factors, while CP content was found to be a predisposing factor for aerobic stability. Surprisingly, both porosity and density were determined to be protective factors. Porosity is directly related to organic matter and, thus, to FHM’s DM, ultimately acting as a protective factor. This could explain the unexpected result of porosity. However, it is worth noting that the porosity values for both stable and unstable samples were found to be rather similar.

### Predictive models

The results from the Lm-AIC models cross-validated between 2016 and 2022 and calculated to predict the DMloss and the FQI, are reported in Table 7. The pre-ensiled traits weaklly predicted the DMloss. Notably, the DM and the ADF contents favoured higher losses, while the CP, the lignin, and the starch contents preserved from losses. Moreover, the DM, the EE, the ADF, the lignin, the WSC, and the starch content in maize plants had a negative coefficient for the FQI, but starch and lignin had the highest negative coefficient, while the CP had the highest positive coefficient. However, the adjusted R^2^ was very poor for DMloss regression, while the adjusted R^2^ and the ratio of performance deviation (**RPD**) were slightly higher for FQI.

**Table 7:** The regression coefficient and metrics from the multivariable linear model (Lm) with variable selection based on Akaike’s information criterion (Lm-AIC) in the backward direction cross-validated between 2016 and 2022 to predict dry matter loss (DM loss) and the silages’ fermentative quality index (FQI) in accordance with maize’s pre-ensiled traits.

												Training set					Validation set		
Estimated	Year	Intercept	DM	Ash	CP	EE	aNDF	ADF	Lignin	WSC	Starch	Adjusted R^2^	MSE	RMSE	RPD	AIC	R^2^	MSE	RMSE
**DM loss**	2016	-23.8	0.34	3.86	-1.64	-2.67		1.48	-2.30	0.08	-4.39	0.33	23.6	4.86	1.22	3087	0.19	35.3	5.95
**DM loss**	2017	-27.2	0.23	4.09	-1.66		0.17	1.03	-1.55			0.26	24.7	4.97	1.16	4228	0.17	9.64	3.10
**DM loss**	2018	-33.3	0.26	3.60	-1.59		0.30	1.03	-1.51		1.64	0.28	4.86	652	0.01	4282	0.22	18.6	4.31
**DM loss**	2019	-16.0		7.73	-3.22	-1.23		0.72	-1.34	0.34		0.35	21.5	4.63	1.07	3272	0.00	36.5	6.05
**DM loss**	2020	-8.2	0.12	2.01	-1.02			0.97	-1.59	-0.14	-0.98	0.27	17.4	4.17	1.17	3894	0.13	101	10.0
**DM loss**	2021	-28.5	0.21	4.28	-1.59	-0.79	0.20	0.92	-1.08	0.09		0.28	22.8	4.78	1.18	4539	0.39	27.0	5.20
**DM loss**	2022	-30.6	0.18	4.31	-1.54	-1.13	0.31	0.71		0.12	1.18	0.27	22.7	4.77	1.17	4569	0.40	40.0	6.32
	Average	-24.0	0.22	4.27	-1.75	-1.46	0.25	0.98	-1.56	0.10	-0.64	0.29	19.6	97.2	1.00	3982	0.21	38.3	5.85
**FQI**	2016	129	-1.52	-6.83	5.31			-1.54	-5.43	-0.13	4.72	0.54	63.0	7.94	1.48	4044	0.33	121	11.0
**FQI**	2017	136	-1.24	-1.94	3.42	-3.68		-1.25	-6.04	-0.26	-11.81	0.51	55.4	7.44	1.43	5292	0.15	129	11.4
**FQI**	2018	132	-1.19	-5.65	4.26	-2.02		-1.10	-4.34	-0.35	-6.47	0.45	63.0	7.94	1.35	5610	0.37	60.0	7.75
**FQI**	2019	92.9	-0.81	-6.33	6.02	-6.91	4.45	-7.81	-5.71	-0.66		0.53	58.0	7.61	1.15	4333	0.03	263	16.2
**FQI**	2020	106	-1.22	-4.49	4.78	-3.09	0.39	-1.25	-5.49		-5.40	0.44	62.2	7.89	1.34	5631	0.16	67.4	8.21
**FQI**	2021	101	-1.07	-4.37	5.05	-2.22		-0.57	-5.48	-0.10	-5.89	0.45	52.7	7.26	1.35	5749	0.35	461	21.5
**FQI**	2022	119	-1.18	-3.83	5.26	-3.98		-1.07	-5.30	-0.19	-7.57	0.49	60.5	7.78	1.40	5999	0.36	176	13.3
	Average	116	-1.18	-4.78	4.87	-3.65	2.42	-2.08	-5.40	-0.28	-5.40	0.49	59.3	7.69	1.36	5237	0.25	182	12.8

DM = dry matter; CP = crude protein; EE = ether extract; WSC = water-soluble carbohydrate; aNDF = alfa-amylase neutral detergent fibre; ADF = acid detergent fibre; DMloss = dry matter loss; FQI = fermentative quality index; MSE = mean square error; RMSE = root mean square error; RPD = ratio of performance deviation.

A Cox-AIC model considering multiple pre-ensiled maize chemical traits was computed and cross-validated by year to predict the event of aerobic instability (Table 8). The model exhibited a higher Somer’s Dxy concordance index of 0.44, with R^2^ exceeding 0.48, except for the validation in 2022, where values of 0.39 and 0.38 were observed for the training and testing sets, respectively. These reported metrics were deemed sufficient for the screening process. Additionally, the VIF indicated inappropriate values for ash, aNDF, and ADF in the 2021 validation, while the P-value for the global Schoenfeld residuals was below 0.05 for validations in 2018 and 2019. Notably, the Cox-AIC algorithm selected aNDF for all years, with lignin showcasing the role of the fibre fraction in estimating aerobic stability, excluding 2019. WSC and starch were prominently selected except in 2021, with EE in 2018, 2020, and 2021, and DM exclusively selected in 2020.

**Table 8:** The multivariable Cox-AIC model to predict the event of aerobic instability by the pre-ensiled traits. The models were cross-validated by year.

	HR	HR, 95% C.I.	Schoenfeld residuals (P)	VIF	Training^1^	Testing
Validation for 2018						
**Crude Protein (g/kg of DM)**	2.14	1.47–3.12	0.03	4.22		
**Ether Extract (g/kg of DM)**	2.75	0.80–9.50	0.51	7.88		
**aNDF (g/kg of DM)**	0.75	0.65–0.88	0.03	3.21		
**Lignin (g/kg of DM)**	4.86	1.9–12.45	0.12	1.70		
**WSC (g/kg of DM)**	1.24	0.99–1.55	0.54	5.00		
**Starch (g/kg of DM)**	0.79	0.69–0.90	0.16	3.63		
**Schoenfeld residuals (Global P)**			0.02			
**R** ^**2**^					0.48	0.48
**Somer’s Dxy concordance index**					0.36	0.36
Validation for 2019						
**Crude Protein (g/kg of DM)**	1.84	1.30–2.62	0.03	2.95		
**aNDF (g/kg of DM)**	0.64	0.53–0.80	0.04	4.26		
**WSC (g/kg of DM)**	1.25	1.06–1.46	0.44	2.10		
**Starch (g/kg of DM)**	0.78	0.67–0.90	0.23	3.73		
**Schoenfeld residuals (Global P)**			0.004			
**R** ^**2**^					0.58	0.58
**Somer’s Dxy concordance index**					0.47	0.47
Validation for 2020						
**Dry Matter (DM, g/kg)**	0.76	0.68–0.85	0.84	6.69		
**Ether Extract (g/kg of DM)**	4.54	1.27–16.2	0.6	6.78		
**aNDF (g/kg of DM)**	0.72	0.57–0.90	0.49	4.24		
**Lignin (g/kg of DM)**	0.12	0.03–0.59	0.23	4.75		
**Starch (g/kg of DM)**	0.83	0.71–0.97	0.51	3.92		
**Schoenfeld residuals (Global P)**			0.51			
**R** ^**2**^					0.53	0.53
**Somer’s Dxy concordance index**					0.48	0.48
Validation for 2021						
**Ash (g/kg of DM)**	15.6	4.80–51.4	0.06	12.6		
**Ether Extract (g/kg of DM)**	4.91	1.18–20.5	0.05	7.69		
**aNDF (g/kg of DM)**	1.46	0.88–2.42	0.34	32.0		
**ADF (g/kg of DM)**	0.40	0.19–0.85	0.49	32.1		
**Lignin (g/kg of DM)**	7.88	0.97–63.8	0.05	5.64		
**Schoenfeld residuals (Global P)**			0.26			
**R** ^**2**^					0.55	0.55
**Somer’s Dxy concordance index**					0.32	0.32
Validation for 2022						
**Ash (g/kg of DM)**	27.5	4.3–177	0.13	3.08		
**aNDF (g/kg of DM)**	0.69	0.57–0.80	0.06	3.78		
**Lignin (g/kg of DM)**	10.6	2.60–43.3	0.31	2.70		
**WSC (g/kg of DM)**	1.50	1.16–1.95	0.16	1.17		
**Starch (g/kg of DM)**						
**Schoenfeld residuals (Global P)**			0.07			
**R** ^**2**^					0.39	0.38
**Somer’s Dxy concordance index**					0.23	0.23

^1^ bootstrapped 10000; VIF = variable inflation factor; aNDF = α-amylase neutral detergent fibre; ADF = acid detergent fibre.

## Discussion

### Exploratory data analysis

Corn silage plays a critical role in dairy production, serving as a significant component of dairy cow diets. It functions as a high-energy feed source, promoting increased milk production due to its high starch content. Furthermore, the fibre content supports healthy rumen function, facilitating efficient digestion and nutrient absorption [56]. Including corn silage in rations helps maintain nutritional balance for dairy cows, threrby providing them with a consistent and high-quality feed option all year round. However, challenges may arise as corn silages need to be regularly monitored throughout the year, requiring adjustments to ration formulations due to variations in nutritive value. Therefore, strategies are required to enhance the nutritive value of corn silage and minimize nutrient losses during storage [57]. Lb is a heterofermentative microbial inoculant used to convert lactic acid into acetic acid, which has antifungal activity, suppresses yeast and mould propagation, and improves aerobic stability [58]. However, the choice of the ensiling method is reported in the literature to impact the findings of the studies, thereby leading to different responses [59].

To bridge this knowledge gap, an analysis was conducted on data from various maize hybrids sown in diverse pedoclimatic conditions. The maize was harvested over six years at varying stages of plant maturity and tested with different inoculant mixtures and laboratory ensiling methods.

With regard to the PCA approach, and according to Kaiser’s criterion, only the first 5 PCs had eigenvalues > 1 and are adapted to be used for further analisys [60]. Indeed, one common application of PCA is to create scatter plots of the first two PCs. This helps visualise and distinguish samples from different groups or categories in a data set. By plotting data points in this reduced-dimensional space, it can be possible to observe patterns, clusters, or separations among the groups, which can provide valuable insights for further analysis or classification. Interesting patterns emerge when plotting the data set samples in the first two PC plot spaces and differentiating them by year, ensiling method, and inoculant. Comparing pre- and post-ensiled PC-1 and PC-2 scatter plots, it appears that most of the variability is in PC-2 for the pre-ensiled plot (with a partial exception for 2019 and 2022). The PCA plots (Fig 1A and 1B) reveal partial segregation of samples in pre- and post-ensile data sets, primarily influenced by the years; this trend was mirrored in PC2. The fluctuations in trial conditions, combined with climatic factors—such as temperature, precipitation, and sunshine hours—may contribute to the variability in maize output [61]. In particular, the literature indicates that diverse growing and harvesting conditions or chop lengths can affect silage attributes among years, which could have led to opposite treatment effects in processed corn silage across different years. [3]. For example, in Fig 1C, it is evident that maize ensiled in buckets or vacuum bags exhibits separation in terms of the first two PCs calculated from the post-ensiled traits. However, when examining the data from 2019, the ensiling methods of buckets and vacuum bags applied to the same samples appear to overlap in the first two principal components. Therefore, in line with recent research, the findings suggest no evident differences between plastic buckets or vacuum-sealed bags in terms of fermentative profiles [60]. Therefore, most of the differences may be attributable to the seasonal effect.

The squared cosine is reported in Table 2, and it is evident that PC1 is predominantly linked to the proximate composition of maize silage, ethanol, lactic acid, and acetic acid. Conversely, PC2 is closely related to pH, mannitol, ethanol, and propionic acid. This visual analysis suggests that variations associated with the different years (stirred on PC2) of the trials likely play a role in influencing the fermentation processes in maize silage. These findings highlight the importance of considering the seasonal aspects when assessing silage quality and associated factors. However, since there is no strong segregation along PC1, which is associated with proximate composition, and all samples appear to belong to the same population, it can be inferred that there is no compelling evidence that indicates differences in the proximate composition of silage attributed to the effects of different years, inoculants, or ensiling methods.

These findings are further validated by the confusion matrix, which is based on k-means clusters and compared across various factors, such as the ensiling method, the use of inoculant, sealing delay, inoculant dose, and the years of the trial, as presented in Table 3. Across all pairs, the MCC remained close to zero (lower than 0.3 in absolute terms). The sensitivity was consistently lower than 0.5, except for the bags in cluster 1, while the accuracy achieved acceptable values only when paired with a specificity greater than 0.8. Once again, these results emphasize that none of the tested factors can independently explain a significant proportion of the cluster’s variability. Determining the optimal time for harvesting maize for whole plant silage poses challenges for farmers. The choice of appropriate plant maturity is crucial for achieving high DM yields [61]. The period from two-thirds of the milk line to the black layer stages is when the yield of the DM is maximized. The moisture, aNDF, and ADF content in FHM decreases with the maturity stage at harvest [62]. Further, previous literature has reported that WSC content decreases from the early dent to the blackline stage due to starch accumulation [25, 63]. WSC plays a vital role in the fermentative pattern of silage, where LAB converts them into organic acids, primarily lactic acid, under anaerobic conditions. This process leads to a decrease in pH, preserving forage from spoilage caused by microorganisms [25]. In our findings, for FHM, the relationships between DM and other chemical traits are not strictly linear. Notably, WSC exhibits an unexpected and uncorrelated trend with DM content (S2 Fig).

Moreover, Table 4 reveals that the average DM at harvest was similar in samples analysed from 2016 to 2020, but WSC indicates considerable differences. These variations might be due to an unknown, probably non-linear, relationship between DM at harvest and plant maturity, likely influenced by climatic and environmental conditions, including the characteristics of different maize hybrids. Indeed, on average, aNDF and ADF decrease with higher DM classes, probably as a consequence of the higher starch content, which acts as a diluent for remaining traits.

On the contrary, aNDF and ADF increase with the last maturity stages. Explaining this pattern is challenging, but it is likely influenced by external conditions and by the use of different maize hybrids in various trials. Interestingly, the composition of FHM does not reveal differences at the input field level when the DM at harvest is similar. These findings suggest that other factors may have a predominant influence on the plant composition. For example, the use of different maize hybrids is reported to lead to varied results in plant composition when harvest comes from diverse field conditions [64].

### The effects of the pre-ensiling conditions on the silage’s fermentative traits

The utilization of the year in the ANCOVA model raised concerns. On the one hand, the effect of the year provided valuable insights into the influence of seasonality. On the other hand, as observed in the exploratory data analysis, PCA revealed partial clustering of samples across the years. Additionally, the years under study represent an almost ‘random’ sample of all potential years. Therefore, while a mixed model could be considered, this research also aimed to emphasise the impact of individual years on silage quality outcomes. However, it is important to acknowledge the challenges associated with replicating and defining the year effect. Climatic conditions do not solely drive this effect but also encompass various intangible factors related to operators’ manuality and other unaccounted variables. However, in the present study, the year did not merely indicate a trend but represented a deliberate and scrutinised ‘factor’ in the analysis.

The post-ensiled silage (Table 5) exhibited significant differences from silages collected in different years, thereby displaying higher WSC in FHM, which did not correspond with high FQI; however, these differences agreed with elevated acetic acid content. FQI is positively influenced by lactic acid and negatively influenced by acetic acid, thereby favouring a higher lactic acid to acetic acid ratio, but it is noteworthy that acetic acid plays a crucial role in inhibiting mould growth [65]. Therefore, it can be stated that FQI fails to be utilisable as an evaluable quality index for assessing the silage’s aerobic stability.

The significant variation in the silage’s DM loss over the years cannot be solely attributed to the effect of environmental conditions and FHM composition since, among the years, there was a change in the method utilised to measure DM loss in buckets. However, it has been reported that different growing and harvest conditions and chop lengths among years may have contributed to the processed corn silage having opposite treatment effects among years [46]. Conversely, there wasn’t a significant difference in the means of DM loss in bags and buckets, thereby confirming that the results from the two methods are comparable, as reported in the literature [60]. However, in this regard, the literature is not concordant since it has been reported that the effect of the use of inoculation (i.e. Lb and *Pediococcus pentosaceus*) revealed significant increases in DM recovery, aerobic stability, acetic acid, ethanol, yeasts, and WSC in buckets but not in bags, compared with the control [66].

With regard to the use of inoculants, the present findings align with those of Jia et al. [24], who investigated the effects of using He or Ho inoculants and concluded that Lb, *Lentilactibacillus plantarum*, and *Lentilactibacillus rhamnosus* improved the fermentation quality compared to the C group, which received no inoculation, thereby confirming that these specific LAB species play a beneficial role in promoting desirable fermentation profiles in maize silage. The present results suggest that heterofermentation by Lb inoculation does not significantly impact DM losses in maize silage. However, a meta-analysis indicates that these losses may vary depending on the type of forage, inoculant composition, and dosage [58]. This suggests that further research is required to fully understand the relationship between Lb inoculants and DM losses in maize silage. However, the effect of the Lb on reducing DM loss is exalted using a combination of homolactic or facultative heterolactic bacteria, beginning with doses of 10^5^ up to 10^6^ CFU g^-1^ while using rates ≥ 10^7^ CFU g^-1^ favours aerobic stability [58]. However, the DM loss observed during the ensiling process must not be confused with the DM loss that occurs during the feed-out period. After seven days of aerobic exposure to wheat silage, a tendency (P = 0.15) of higher content of acetic acid revealed a prolonged perdurance of this trait in silage treated with He (0.824% of the DM) as compared to Ho (0.56%of the DM) and control (0.583% of the DM) [21], under the assumption that the role of He is more valuable after the silo opening.

In the present results, while the use of different inoculant doses (HD, SD, and DD) resulted in inconclusive findings, it is worth noting that the differences between SD, HD, and DD in terms of colony-forming units (CFUs) were likely too subtle to reveal a significant impact on fermentation quality. In contrast the literature reports that incremental doses of Lb (i.e. 1 × 10^5^, 5 × 10^5^ and 1 × 10^6^ CFU g^-1^) resulted in a more heterolactic fermentation and improved aerobic stability of the maize silages by increasing the concentration of acetic acid and decreasing the numbers of yeasts; moreover the 5 × 10^5^ CFU g^-1^ was the most effective on maize silages [65]. However, in the present study, the DD was the higher dose used (i.e. 4.04 × 10^5^ CFU g^-1^) was substantially lower than the dose suggested in the literature. Therefore, it is recommended that a logarithmic scale be used to differentiate between inoculant dosages when preparing mixing rations. This approach would enable a more nuanced assessment of the effects of varying inoculant concentrations on silage fermentation [44].

Prolonged exposure to air during the silage storage process can have both positive and negative consequences. On the one hand, moderate delay (less than 6 h) can lead to a reduction in dry matter (DM) losses, thereby confirming the findings by Kim and Adesogan [67]. According to our results, in the literature, it is reported that increasing air exposure (up to 210 min between chopping and sealing) before sealing was recognized to increase fermentation losses, probably because of higher gas production [22]. This is attributed to the increased activity of aerobic microorganisms, which consume certain easily fermentable sugars, thereby minimising the loss of nutrients. On the other hand, prolonged exposure to air can hinder the appropriate fermentation process, thereby producing undesirable by-products and accumulating off-flavors [67]. Therefore, balancing the potential benefits of delayed sealing with the risk of compromising silage quality is crucial.

Our findings partially support a previous study that found no interaction between delay time and inoculation on the lactic acid, acetic acid, and ethanol contents of silage [22]. However, our findings revealed that lactic acid content was positively affected by the use of Ho or He under progressive sealing delay. In contrast with the cited literature, our findings revealed a tendency (P = 0.117) for lactic acid to increase with progressive delay while significantly increasing with the use of inoculants. The reasons underlyng these contrasting results are still unclear. However, it is important to note that the differences observed in the use of inoculants for progressive silo delay are statistically significant, but they do not appear to have any functional relevance.

Harvesting maize at a lower DM class positively influenced the fermentation profile and the FQI while tendentially reducing the pH. This could be attributed to a higher content of WSC in plants harvested at an earlier stage of maturity. Interestingly, DM losses were higher for silages harvested at both lower and higher DM classes. This can be explained by the more vigorous fermentation in lower DM classes, leading to faster sugar depletion with water, carbon dioxide production, and energy consumption. Additionally, silages harvested at higher DM classes may have been inefficiently compacted during storage [46], thereby resulting in lower density and higher porosity, which could enhance aerobic microbial activity and contribute to DM losses.

The use of the inoculants, particularly the Ho, resulted in higher FQI for DM class high compared to the control. These findings are supported by higher lactic acid and lower propionic acid content in Ho and He silages harvested at a high DM class. Moreover, the use of He reduced the DM loss in silages harvested at DM class low and medium by providing effective competition with yeast during the aerobic and fermentation phases [46]. The maturity of corn silage also had an impact on DM loss, which is likely related to the decreased wet-pack density; moreover, it was noted that as maturity advanced, wet-pack density measurements decreased [46]. Pack density in the silo is indeed important for preserving corn silage quality during storage and can help to prevent the growth of aerobic microorganisms and preserve the quality of the silage [68]. The density of the silage, along with its DM content, determines the porosity, which is a measure of the air spaces within the silage mass. A higher porosity enables more air to infiltrate the silage, which can lead to spoilage by aerobic microorganisms [46]. Therefore, the use of inoculants should be relevant in higher DM classes. Conversely, in the present findings, the inoculants did not affect the DM loss (probably because of the inadequate ratio distribution), which could be why the effect was not marked, particularly for DM class high, where it was expected to be more relevant.

The effect of the input field is challenging to explain. Even though all traits revealed significant differences in means, with better FQI and lower DM loss in IFH, the observed values are functionally rather similar among input field levels. Numerous studies have demonstrated the significant influence of plant maturity on fermentative characteristics in both whole-crop oats and maize silages. In fact, Jia et al. [24] specifically highlighted this impact in oat silages. Our findings corroborate those of previous studies, which indicate that the maturity stage of the plant plays a crucial role in influencing the fermentative characteristics of maize silage. This is evident from the decreased acetic acid production and declined FQI observed in silages harvested at later maturity stages compared to earlier stages. This is likely due to the changes in sugar content that occur during maturation. Moreover, silages harvested at rather late stages of maturity have a higher rate of acetic acid production, which could affect the pH and increase the aerobic stability of the silage. Previous studies have documented that maize silages harvested at later stages of maturity—such as the one-third, two-thirds, or black line stages—exhibit a higher proportion of acetic acid production compared to those harvested at the early dent stage [25]. This increased acetic acid production is attributed to the higher starch content in later-maturing maize plants, which provides more substrate for the acetic acid-producing bacteria. Moreover, the lower WSC content in later-maturing maize may also increase pH, thereby further promoting acetic acid production ahead of lactic acid. Similarly, Johnson et al. [4] found that maturity at harvest substantially impacted fermentation characteristics in maize silage compared to inoculation, thereby suggesting that the natural microbial population within the maize plant plays a crucial role in determining the fermentation profile.

The differences between the hybrids employed can be attributed to the plant composition. This variability among the hybrids impacted the FQI, particularly with regard to the levels of lactic and propionic acids. As the mean WSC content in early-flowering (EA) and late-flowering (LA) hybrids is rather similar, the observed disparities in the fermentative profile are likely associated with the variability in pre-ensiled traits among the EA or LA hybrids.

### The effects of the pre-ensiling conditions on silage’s aerobic stability

The Cox proportional hazard regression is a widely used technique for modelling cause-specific hazards in research involving time-to-event survival analysis, such as clinical trials [69] and, in the present study, the aerobic instability assessments in silage [44, 45]. The results from the univariable Cox regression indicate the effect of the season on the aerobic stability of silage, thereby confirming the role of harvest conditions and chop lengths between years [46]. The role of the He was confirmed to be more protective compared to the control group, probably due to its effect occurring during aerobic exposure. Increased delays are a predisposition for aerobic instability, probably because, as discussed earlier, the delay affects the fermentative path consuming WSC for respiration due to the increased activity of aerobic microorganisms and, therefore, leading to lower availability for LAB fermentation. Moreover, earlier harvested maize, compared to later harvests, has both more available WSC and higher humidity, which permits higher pack density, leading to—as previously discussed—prolonged aerobic stability. DM, EE, and starch are the FHM traits that maintain aerobic stability. Noticeably, the mean for aerobic stability and instability were 34.9% and 32.5%, respectively. It is probable that at the DM associated with the stable group, the packing density has been warrantied in the studied silage, and the limits for unreliable adequate density are associated with a DM greater than 35%.

### Predictive models

The use of pre-ensiled traits to predict DM loss and FQI was not very successful. This suggests that other factors—such as seasonality, the use of inoculants, sealing delays, and the choice of maize hybrids—need to be considered in the models. However, for FQI, the pre-ensiled traits were able to explain over 50% of the variability in the data.

Further, even the Cox-AIC models were not very effective in predicting aerobic instability. The fibre composition appeared to have a certain amount of influence, with harvests with higher lignin and lower aNDF content being more aerobically unstable. In the plants, the aNDF tends to be diluted by the increasing starch content in late harvests, while lignin tends to increase in later maturity stages. Therefore, plants harvested at later maturity stages appear to be more aerobically unstable. However, from Table 7, stable silage was found to have less than 35% DM. The aerobic stability effect may reverse at higher DM contents due to a reduced wet-packing density.

As with the LM models, the Cox-AIC models suggest that other factors, such as those mentioned above, should be considered to improve their prediction ability. However, pre-ensiled traits can still be useful for screening purposes or in-field applications, such as sensor-based harvesting systems. This can help to relate the silage quality to the harvest composition and predict the need for further management decisions, such as the use of appropriate inoculants or intensive packing.

Given that the FQI is designed to condense the fermentative profile of silage into a singular metric, it emerges as the most appropriate candidate for serving as a comprehensive indicator that facilitates an understanding of maize fermentative quality. In contrast, DM losses serve as a tangible indicator directly associated with the costs related to the ensiling process. Consequently, these two metrics were incorporated into the predictive models. However, the Cox model was explicitly employed to forecast aerobic stability, a critical aspect alongside DM losses and the fermentative profile (i.e., FQI) in characterizing maize silage within this study. Conversely, although it is theoretically possible to develop models for all fermentative traits (despite the potential overlap with the FQI), doing so would generate a data set that is too voluminous for inclusion in a single manuscript. Finally, while various aspects of maize silage could potentially be considered (such as changes in composition post-ensiling), these are beyond the scope of the present study’s objectives.

## Conclusions

Optimizing silage quality involves balancing three key aspects: minimizing dry DM loss, achieving a desirable fermentation profile, and enhancing aerobic stability. These aspects are influenced by a multitude of factors, and their effects can be interconnected and occasionally contradictory. While certain aspects of silage quality exhibit consistent patterns, such as the use of Ho or He inoculants leading to higher production of lactic and acetic acids, respectively, other aspects remain uncertain, such as the optimal inoculant dosage and the most effective in-lab techniques. Nevertheless, it is crucial to consider the interaction of these factors, as the impact of a single factor can vary depending on its interplay with other factors. This study highlights the significant impact of seasonality, thereby suggesting that regional considerations may also play a role in optimizing silage quality. The predictive models developed in this study demonstrate adequate performance for screening purposes but fall short of providing precise predictions. This emphasises the need for a more comprehensive and extensive data set to develop more accurate models. Merging data from diverse studies into a larger and more informative data set is strongly recommended. This would enable the application of advanced statistical methods, such as machine learning and deep learning, to obtain a deeper understanding of silage quality and develop more reliable prediction models.

## Supporting information

S1 Fig Scatter plot of the first 2 PC (F1 and F2) for samples assigned to the K-means classes of two, three, four, and seven clusters. Ovals represent the 95% confidence interval.
(TIF)

S2 Fig The relationship between fresh maize water-soluble carbohydrates and dry matter content. Scatter plot and linear regression of water-soluble carbohydrates (WSC) as a function of the dry matter (DM) content of freshly harvested maize (FHM).
(TIF)
