# Supplementary figures and images for: A comparative evaluation of maize silage quality under diverse pre-ensiling strategies
Source: PLoS One. 2024 Sep 18;19(9):e0308627. doi: 10.1371/journal.pone.0308627 (PMC11410270; doi:10.1371/journal.pone.0308627)

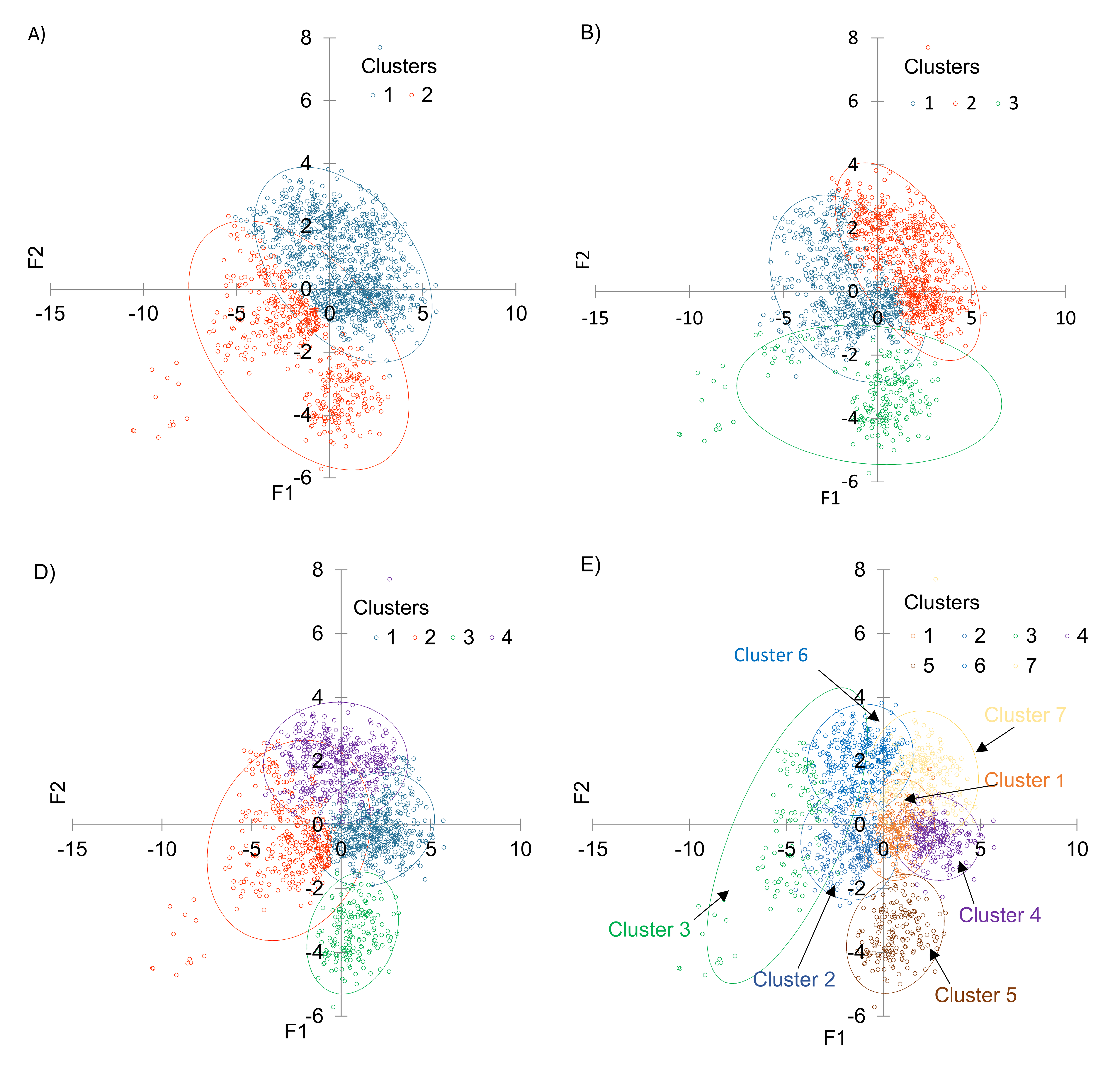

Supplement: S1 Fig — Ovals represent the 95% confidence interval. (TIF) [file pone.0308627.s001.tif]

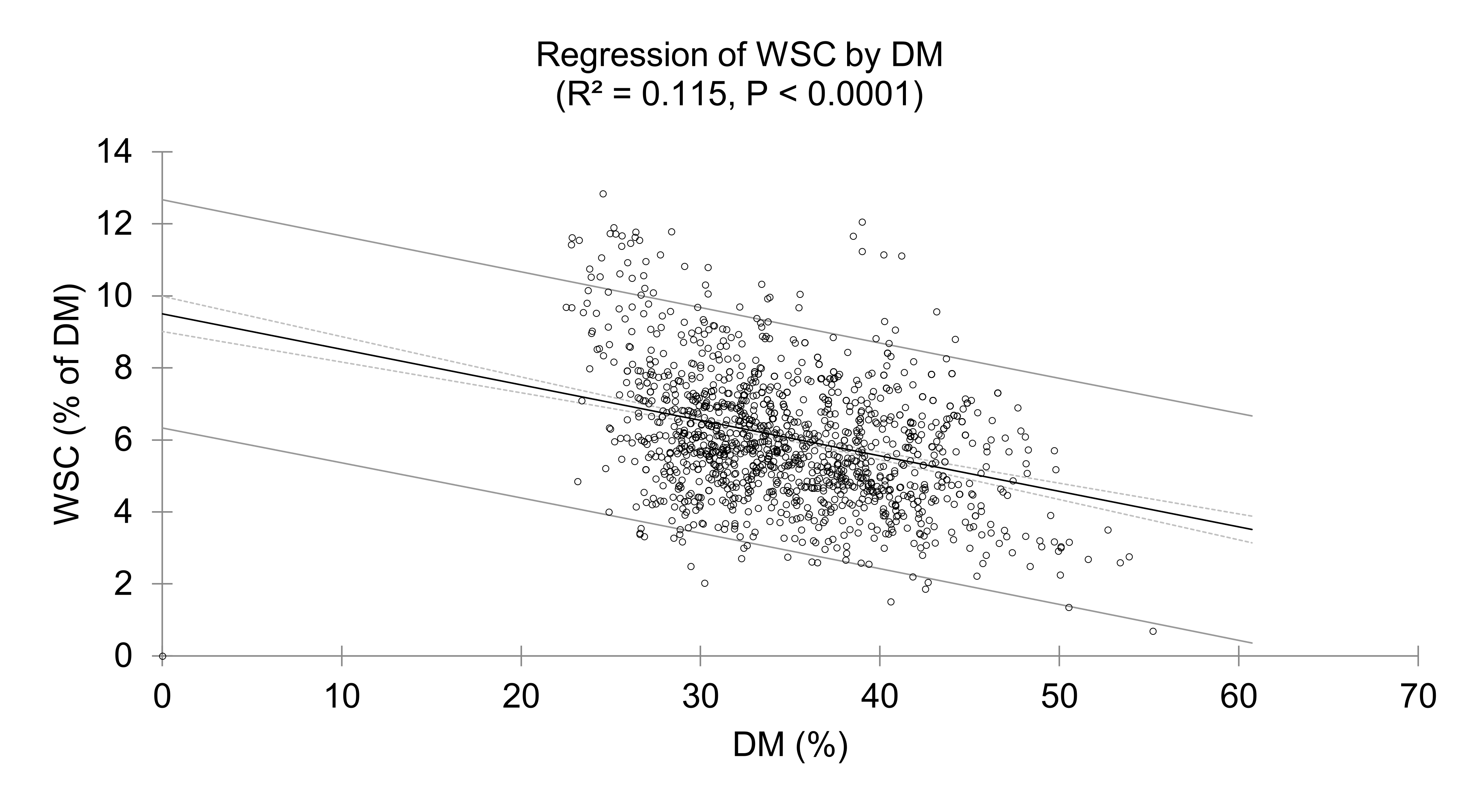

Supplement: S2 Fig — Scatter plot and linear regression of water-soluble carbohydrates (WSC) as a function of the dry matter (DM) content of freshly harvested maize (FHM). (TIF) [file pone.0308627.s002.tif]
